# Effect of maternal obesity on birthweight and neonatal fat mass: A prospective clinical trial

**DOI:** 10.1371/journal.pone.0181307

**Published:** 2017-07-27

**Authors:** Delphine Mitanchez, Sophie Jacqueminet, Jacky Nizard, Marie-Laure Tanguy, Cécile Ciangura, Jean-Marc Lacorte, Céline De Carne, Laurence Foix L’Hélias, Pascale Chavatte-Palmer, Marie-Aline Charles, Marc Dommergues

**Affiliations:** 1 Department of Perinatality, APHP, GHUEP, Armand Trousseau Hospital, Paris, France; 2 Sorbonne Universities, UPMC University Paris 06, Paris, France; 3 Department of Diabetology, Institute of Cardiometabolism and Nutrition (ICAN), APHP, University Hospital Pitié-Salpêtrière, Paris, France; 4 Department of Gynecology and Obstetrics, APHP, University Hospital Pitié-Salpêtrière, Paris, France; 5 Department of Clinical Research, APHP, University Hospital Pitié-Salpêtrière, Paris, France; 6 Endocrine and Oncologic Biochemistry, APHP, University Hospital Pitié-Salpêtrière, Paris, France; 7 UMR BDR, INRA, ENVA, Université Paris Saclay, Jouy en Josas, France; 8 Inserm, U1153, Epidemiology and Biostatistics Sorbonne Paris City Research Centre, Villejuif, France; 9 Univ Paris Descartes, Sorbonne Paris Cité, UMR1153, Paris, France; Centre Hospitalier Universitaire Vaudois, FRANCE

## Abstract

**Objective:**

To discriminate the effect of maternal obesity and gestational diabetes on birth weight and adipose tissue of the newborn.

**Methods:**

Normal BMI women (group N, n = 243; 18.5≤ BMI<25 kg/m^2^) and obese women (group Ob, n = 253; BMI≥30 kg/m^2^) were recruited in a prospective study between 15 and 18 weeks of gestation. All women were submitted to a 75g oral glucose tolerance test in the second and third trimester. First trimester fasting blood glucose was also obtained from Ob women. All women with one measurement above normal values were considered positive for gestational diabetes and first treated by dietary intervention. When dietary measures were not efficient, they were treated by insulin. Neonatal anthropometrics, sum of skinfolds and cord serum hormones were measured.

**Results:**

222 N and 226 Ob mothers and their newborns were included in the analysis. Diabetes was diagnosed in 20% and 45.2% of N and Ob women, respectively. Birth weight was not statistically different between groups (boys: 3456g±433 and 3392g±463; girls: 3316g±402 and 3391g±408 for N and Ob, respectively). Multivariate analysis demonstrated that skinfold thickness and serum leptin concentrations were significantly increased in girls born to women with obesity (18.0mm±0.6 versus 19.7mm±0.5, p = 0.004 and 11.3ng/mL±1.0 versus 15.3ng/mL±1.0, p = 0.02), but not in boys (18.4mm±0.6 versus 18.5mm±0.5, p = 0.9 and 9.3ng/mL±1.0 versus 9.0ng/mL±1.0, p = 0.9). Based on data from 136 N and 124 Ob women, maternal insulin resistance at 37 weeks was also positively related to skinfold in girls, only, with a 1-point increase in HOMA-IR corresponding to a 0.33mm±0.08 increase in skinfold (p<0.0001).

**Conclusions:**

Regardless of gestational diabetes, maternal obesity and insulin resistance were associated with increased adiposity in girls only. Persistence of this sexual dimorphism remains to be explored during infancy.

## Introduction

Many countries currently face an increasing prevalence of obesity and related medical complications. Maternal pre-pregnancy body mass index (BMI) ≥30 kg/m^2^ is associated with increased rates of many complications during pregnancy for the mother, the fetus and the neonate [1], including fetal malformations, perinatal mortality [2, 3] and fetal overgrowth with subsequent neonatal macrosomia [4]. The relationship between maternal obesity and macrosomia is well documented, but the specific effect of obesity versus gestational diabetes remains unclear. Indeed, a recent meta-analysis concluded that maternal obesity is associated with excessive fetal growth [5], but there was substantial clinical heterogeneity between the studies included in this meta-analysis, notably concerning the method for recording maternal weight and the inclusion of diabetic mothers.

The primary objective of this study was to determine the relative contribution of maternal obesity and controlled gestational diabetes on infant skinfold, as well as birthweight. To this effect, we designed a prospective exposure-matched cohort study comparing neonates born to women with normal pregestational BMI (18.5 ≤ BMI < 25 kg/m^2^) and neonates born to obese women with pregestational BMI ≥ 30 kg/m^2^. Screening and subsequent treatment for gestational diabetes were enhanced in order to minimize the potential effect of maternal hyperglycemia on neonatal anthropometrics.

## Methods

This prospective exposure-matched cohort study was registered as clinical trial registration number: NCT02681588, ClinicalTrials.gov. The trial began before the clinical trial registration was required for this type of observational study. At that time, in 2010, the sponsor did not do it systematically. When this became required, compliance was made for all trials and new trials were registered before recruiting the first participant. The authors confirm that all ongoing and related trials for this intervention are registered. The study received the approval of the Ile-de-France ethics committee on November 18, 2009 (CPP: Committee of Protection of the People—n°79–09). All participants gave written informed consent. Patients were recruited in two Parisian hospitals (centers 1 and 2) before 18 weeks of gestation between August 31, 2010 and March 19, 2013. The delivery of the last patient included in the study occurred on September 23, 2013 and the end of follow-up for the baby was on October 21, 2013. Inclusion criteria were pregestational BMI ≥ 30 kg/m^2^ (obese mothers) or 18.5 ≤ BMI < 25 kg/m^2^ (normal weight mothers), maternal age 18 years or greater and below 41 years, singleton pregnancy. Exclusion criteria were: initiation of antenatal care after 18 weeks, known type 1 or type 2 diabetes, obesity due to a genetic disorder or secondary to intracranial tumor or radiotherapy, bariatric surgery, chronic diseases other than obesity and non-fluency in French.

Gestational age was determined by ultrasound between 6 and 13 weeks for all patients.

As soon as a woman with BMI≥30 kg/m^2^ was included, a pregnant woman with 18.5≤ BMI< 25 kg/m^2^ was recruited based on the following matching criteria: age ± 5 years, nulliparity or multiparity, gestational age at inclusion ± 4 weeks. Written informed consent was obtained by the study participants.

Prenatal care and timing of delivery were determined by local standards of care, with the exception of screening for glycemic status. After initial booking, women were examined monthly by a doctor or a midwife. Bodyweight, blood pressure and proteinuria (assessed by a dipstick test) were recorded. Fetal ultrasound examination was also performed for all women at approximately 22 and 32 weeks of pregnancy.

### Maternal characteristics

Total pregnancy weight gain was calculated based on the difference between the last weight measured within one month before delivery and the weight measured before pregnancy. Weight gain was classified according to the guidelines issued by the Institute of Medicine (IOM) (http://www.nationalacademies.org). All patients received the general pregnancy dietary recommendations according to the French national program of nutrition and health recommendations (http://www.inpes.santepubliquefrance.fr). Weight gain targets were explained but no specific intervention for weight management during pregnancy was proposed. Recommended weight gain was 11 to 16 kg for women of normal weight and 5 to 9 kg for women with obesity.

Pregnancy induced hypertension was defined as systolic blood pressure >140 mm Hg and diastolic blood pressure > 90 mm Hg. Preeclampsia was defined as pregnancy induced hypertension and proteinuria ≥ 0.3 g/24 hours or albuminuria/creatininuria ratio ≥ 0.02 g/mmol. Preterm delivery was defined as delivery before 37 weeks.

Fetal losses included miscarriage, termination of pregnancy and intrauterine fetal death.

### Gestational diabetes

Screening for gestational diabetes was based on our standard procedure including a fasting blood glucose (FBG) in the first trimester for women with BMI ≥ 30 kg/m^2^, and a 75g oral glucose tolerance test (OGTT) between 24–28 weeks regardless of maternal BMI. A limited number of women with 18.5≤ BMI< 25 kg/m^2^ also had a first trimester FBG prior to booking. In addition to this routine practice, women were also screened for gestational diabetes at 32 weeks by performing a 75g-OGTT, regardless of maternal BMI.

Gestational diabetes was defined according to the thresholds published by the International Association of Diabetes and Pregnancy Study Groups (IADPSG) [6]: for the diagnosis of gestational diabete, one or more of the following values for glucose concentration after a 75g OGTT must be equal or exceed 5.1 mmol/L for FBG, 10 mmol/L for 1-h plasma glucose and 8.5 mmol/L for 2-h plasma glucose. The same blood glucose thresholds were used in the second and the third trimester. All women with one measurement above normal values were considered positive for gestational diabetes and treated according to the same protocol.

If gestational diabetes was diagnosed, patients were referred to one of the two diabetologists involved in the study. The first line treatment was dietary intervention with a standard 1800 kilocalories daily meal plan divided into three meals and snacks. All patients were assigned a memory reflectance meter and were instructed by the diabetologist on glycemia self-monitoring (6 times/daily: fasting and 2h-postprandial). The objectives of treatment were to maintain fasting glucose level <0.90 g/L and post-prandial level <1.2 g/L. Diabetologists evaluated the quality of the glycemic control every two weeks. Patients who were unable to achieve the established goals by dietary control only were prescribed insulin treatment after two weeks of failed dietary therapy.

### Neonatal characteristics

Neonates were born between January 14, 2011 and September 23, 2013. After delivery, neonates received routine care. Birth weight was measured immediately after birth without diaper, using a calibrated electronic scale. Length was measured with a stadiometer, ponderal index was calculated for all the patients (weight/length^3^x100, g/cm^3^). Skinfold thickness was measured with a skinfold caliper (Harpenden, Baty, UK) within 72h of delivery. To ensure accuracy and reproducibility of the measurement, the pediatricians in both centers attended a dedicated training course. Measurement of skinfold thickness was done at four sites: triceps, biceps, suprailiac and subscapular, according to the method described by Schmelzle [7]. If two measurements differed by more than 0.5 mm, at least one extra measurement was taken until two similar measurements were obtained. The average of the two closest measurements at each site was calculated and the sum of the values at the four sites was done. The sum of skinfolds was used for the present study. Skinfolds were not measured in preterm infants (less than 37 weeks).

Weight and length Z-scores were determined using standard neonatal weight and length curves established in the French population taking into account gestational age and the sex of the neonate and up-dated in 2005 (http://www.audipog.net/courbes_morpho.php). Large for gestational age was defined as birth weight > 90^th^ percentile and small for gestational age as birth weight < 10^th^ percentile.

Placental weight was obtained immediately after delivery using a calibrated electronic scale.

### Biological assays

At 37 weeks, maternal FBG and insulinemia were assayed. HOMA-IR was calculated using the following formula: HOMA-I = (fasting plasma insulin (mUI/l) ×fasting blood glucose (mmol/L))/22.5.

On admission to the delivery suite, maternal blood was collected for HbA1c and hormones assays. HbA1c was measured immediately and the serum was aliquoted and immediately frozen at -80°C for subsequent leptin and adiponectin assay. After delivery, glycemia was measured immediately in cord blood, serum aliquots were stored immediately at -80°C until leptin and C-peptide assays were performed.

Leptin and total adiponectin were measured by ELISA (leptin, Biovendor, Eurobio, Les Ulis, France; Total adiponectin, ALPCO, Eurobio, Les Ulis France), according to manufacturer’s instructions with inter-assay coefficient variation of 7.2% and 6.2% respectively. C-peptide was measured by an automated chemiluminescent immunoassay (Liaison XL, Diasorin, France). All assays were performed at the end of the recruitment period except for C-peptide quantifications that were performed every 6 months in order to avoid degradation of the molecule.

### Statistical analysis

The sample size was based on the results of a study that compared skinfold thickness between two groups of women (BMI ≥ 25 and < 25 kg/m^2^)^.^ The size effect was 0.42 [8]. The required sample size was calculated to detect an effect size of 0.3 for neonatal sum of skinfolds. With an estimated rate of women excluded from analysis (premature births, fatal issues and lost to follow up) of 24% and a two-sided type-1 error of 5%, the inclusion of 618 women provided a power of 90% based on a one-way analysis of variance. The recruitment was lower than expected and only 496 women were included at the planned study end date. However, with 496 women included, and since the rate of women excluded from the analysis was lower than expected, the final power of the study remained acceptable (82%).

Maternal and neonatal characteristics according to maternal BMI (Table 1) were compared using student’s t-tests or analyses of variance for continuous data and chi-squares or Fisher’s exact tests for categorical data. Comparisons between non-diabetic obese women and diabetic obese women or non-diabetic women in the N group (Table 2) were performed with a Dunnett's test to address for multiplicity. A stepwise multivariate analysis of variance was performed to identify factors associated with sum of skinfolds, weight Z-score, leptin concentration and placental weight. The parameters introduced in the model were, in addition to maternal obesity and gestational diabetes (yes vs no), center, age at inclusion, ethnicity (Caucasian vs other), gestational weight gain (within, more or less than recommended), present smoking status, parity, pregnancy induced hypertension, HbA1c at delivery, gestational age at birth, newborn’s sex.

**Table 1:** Maternal and neonatal characteristics according to maternal BMI. Data are expressed as mean [SD] or N (%).

	18.5≤ BMI<25 kg/m^2^	BMI≥30 kg/m^2^	
**Maternal characteristics**	**N = 222**	**N = 226**	**P-value**
**Age** (years)	30.9 [4]	30.8 [4.7]	0.7
**Primiparity n (%)**	82 (36.9)	89 (39.4)	0.6
**Gestational age at inclusion** (wks)	14.8 [2.1]	14.8 [2.3]	0.9
**BMI (kg/m**^**2**^**)**			
*Before pregnancy*	21.3 [1.7]	34.7 [4.6]	**<0.0001**
*At inclusion*	22.4 [1.9]	35.5 [4.4]	**<0.0001**
**Education n(%)**			**<0.001**
No diploma	8 (3.6)	26 (11.6)	
Secondary school	18 (8.2)	44 (19.6)	
High school diploma	25 (11.4)	50 (22.2)	
Higher education	169 (76.8)	105 (46.7)	
**Ethnicity n (%)**			**<0.001**
White European	154 (69.7)	76 (33.9)	
Northern Africa	18 (8.1)	52 (23.2)	
Sub Saharan Africa	31 (14)	89 (39.7)	
Other	18 (8.1)	7 (3.1)	
**Gestational hypertension n (%)**	2 (0.9)	14 (6.3)	**0.002**
**Caesarian section n (%)**	36 (16.3)	102 (45.1)	**<0.001**
**Smoking during pregnancy n (%)**	22 (10.1)	22 (9.8)	0.9
**Alcohol use n (%)**	9 (4.1)	5 (2.2)	0.3
**Gestational weight gain (kg)**	13.5 [4.2]	8.2 [7.4]	**<0.0001**
Within recommended range n (%)	90 (42.7)	56 (26.5)	**<0.01**
More than recommended n (%)	52 (24.6)	96 (45.5)	
Less than recommended n (%)	69 (32.7)	59 (28.0)	
**Gestational diabetes n (%)**	41 (20)	99 (45.2)	**<0.0001**
Insulin treatment	8 (21.1)	49 (52.1)	
Gestational age at diagnosis n (%)			
< 18 weeks	4 (9.8)	49 (49.5)	
24–28 weeks	13 (31.7)	36 (36.4)	
32 weeks	24 (58.5)	14 (14.1)	
**Maternal biological data**			
Glycaemia at 37 weeks (mmol/L)	4.1 [0.5]	4.3 [0.5]	**<0.01**
Insulinemia at 37 weeks (mU/mL)	10.7 [8.4]	19.6 [11.6]	**<0.0001**
HOMA-IR at 37 weeks	2.0 [1.8]	3.8 [2.6]	**<0.0001**
HbA1c at delivery (%)	5.4 [0.3]	5.5 [0.4]	**<0.0001**
Leptin at delivery (ng/mL)	23.0 [14.2]	48.7 [25.3]	**<0.0001**
Adiponectin at delivery (μg/mL)	6.0 [2.32]	5.0 [2.5]	**<0.0001**
**Neonatal characteristics**	**N = 222**	**N = 226**	
Gestational age (weeks)	39.8 [1.1]	39.6 [1.1]	0.09
Boys n (%)	117 (53)	103 (45)	0.13
**Birthweight (g)**			
Boys	3456 [433]	3392 [463]	0.24
Girls	3316 [402]	3391[408]	0.43
Weight Z-score	0.01 [0.95]	0.08 [0.96]	0.4
Ponderal index (g/cm^3^)	2.75 [0.24]	2.78 [0.28]	0.2
Sum of skinfolds (mm)	17.9 [3.3]	19.4 [3.9]	**<0.0001**
**Cord blood biological data**			
Glycemia (mmol/L)	4.2 [0.92]	4.2 [1.0]	0.5
Leptin (ng/ml)	9.7 [8.8]	12.6 [10.1]	**<0.01**
C-peptide (ng/mL)	0.77 [0.7]	0.96 [0.7]	**0.04**
***Boys***			
Sum of skinfolds (mm)	18.1 [3.5]	18.8 [4.2]	0.20
Leptin (ng/ml)	8.4 [8]	8.9 [6.5]	0.7
Placenta weight (g)	570 [132]	602 [150]	0.21
***Girls***			
Sum of skinfolds (mm)	17.8 [3.1]	19.9 [3.6]	**<0.0001**
Leptin (ng/ml)	11.1 [9.4]	15.6 [11.34]	**<0.01**
Placenta weight (g)	549 [122]	609 [126]	**<0.01**

**Table 2:** Maternal and neonatal characteristics according to maternal BMI and to diabetic status. Obese non-diabetic women were compared to obese diabetic women (a and b superscripts) and to non-obese non-diabetic women (c and d superscripts). Data are expressed as mean [SD] or N (%).

	18.5≤ BMI<25 kg/m^2^		BMI≥30 kg/m^2^	
	No diabetes	Gestational diabetes	No diabetes	Gestational diabetes
	N = 164	N = 41	N = 120	N = 99
***BMI kg/m***^***2***^	21.4 [1.6]^c^	21 [1.9]	34.5 [4.6]^d^	35 [4.5]
***Gestational weight gain (kg)***	13.3 [3.9]^c^	13.6 [5.7]	9.6 [6.7]^a^^,^^d^	6.2 [8.1]^b^
***Maternal metabolic data***				
Insulinemia at 37 weeks (mU/mL)	10.8 [9.2]^c^	10.2 [4.5]	19.4 [9.8]^d^	20.2 [14.1]
HOMA-IR at 37 weeks	2 [2.0]^c^	1.9 [0.9]	3.7 [2.0]^d^	4.1 [3.4]
HbA1c at delivery %	5.3 [0.3]^c^	5.5 [0.3]	5.5 [0.4]^d^	5.6 [0.5]
Leptin at delivery (mg/mL)	23.5 [13.8]^c^	22.4 [16.7]	53.5 [27.2]^a^^,^^d^	43.6 [22.9]^b^
Adiponectin at delivery (μg/mL)	5.8 [2.1]^c^	6 [2.3]	5 [2.9]^d^	4.9 [1.9]
***Neonatal anthropometrics***				
Birth weight (g)	3400 [433]	3344 [379]	3361 [443]	3430 [420]
Weight Z-score	0.01 [0.94]	0.02 [1.02]	-0.03 [0.97]	0.21 [0.96]
Large for gestational age	17 (10.4)	2 (4.9)	11 (9.2)	12 (12.1)
Small for gestational age	15 (9.2)	4 (9.8)	11 (9.2)	8 (8.1)
Sum of skinfolds (mm)	17.8 [3.1]^c^	18.6 [3.7]	19 [3.5]^d^	19.9 [4.4]
***Neonatal metabolic data***				
Glycemia (mmol/L)	4.1 [0.9]	4.4 [1.1]	4.2 [1.0]	4.3 [1.1]
Leptin (ng/mL)	10.2 [9.7]	9.6 [5.5]	12.5 [8.6]	12.5 [11.4]
C-peptide (ng/mL)	0.81 [0.77]	0.7 [0.44]	0.81 [0.47]^a^	1.14 [0.9]^b^
***Placental weight (g)***	562.6 [123]	548.3 [134]	585.4 [130]	632.1 [143]

^a,b^ p<0.05 for comparison between non-diabetic obese women and diabetic obese women.

^c,d^ p<0.05 for comparison between non-diabetic normal weight and non-diabetic obese women.

We tested interactions of sex with obesity, diabetes and HbA1c. When an interaction with sex was significant, pairwise comparisons were made using Tukey’s method to account for multiple comparisons. All tests were two-sided. The alpha level was set at 0.05. Analyses were performed using SAS software V9.3 (SAS Institute, Cary, NC).

## Results

**Fig 1:**
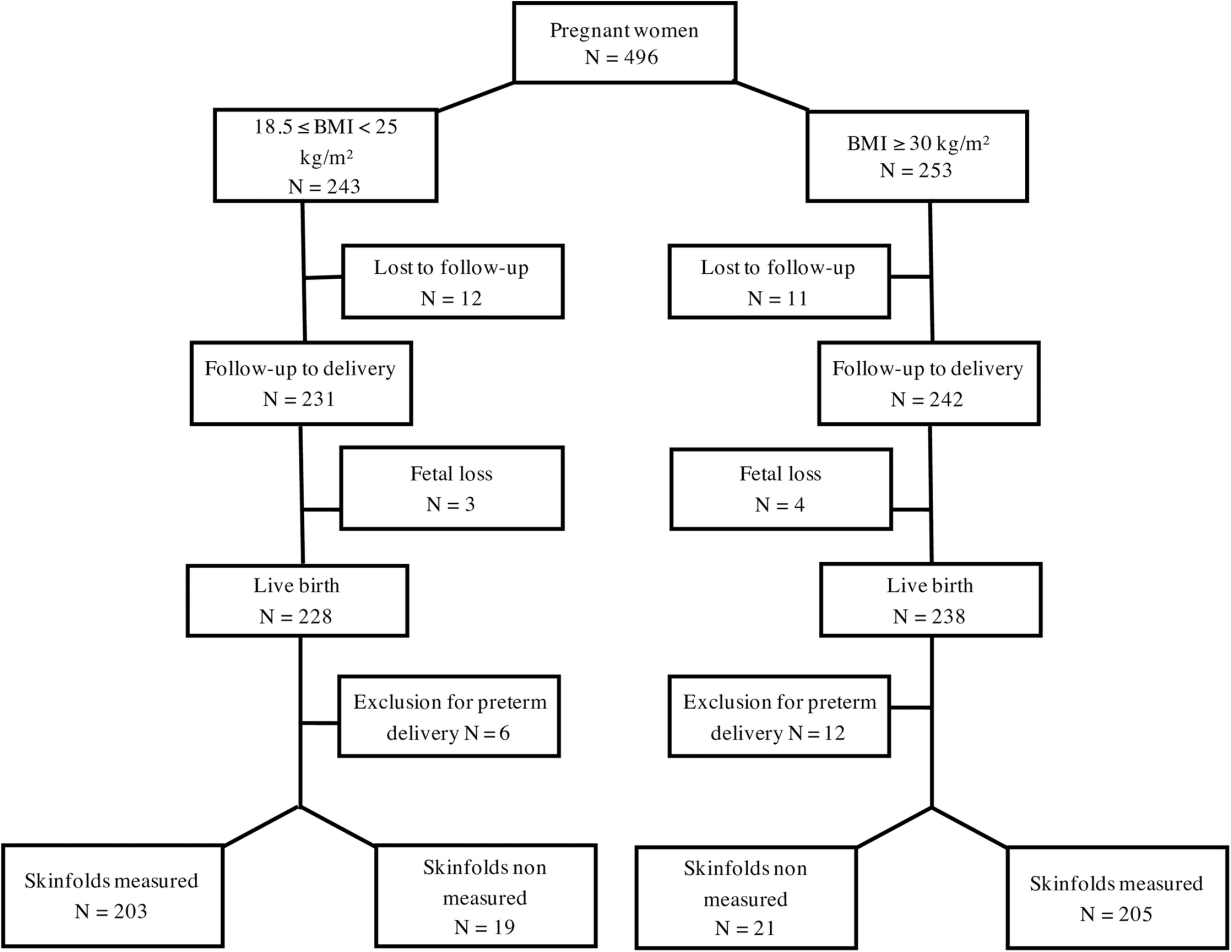
Flow chart of the study. Fetal losses in the group of women of normal weight were due to one termination of pregnancy for growth restriction and two fetal deaths; fetal losses in the group of women with obesity were due to one miscarriage, two terminations of pregnancy for neural tube defect and corpus callosum agenesis and one fetal death.

Four hundred and ninety-six pregnant women were recruited, 353 in center one, 143 in center two. Two hundred and forty-three pregnant women had normal BMI (group N, 18.5≤ BMI<25 kg/m2) and 253 were obese (group Ob, BMI≥30 kg/m^2^). Twelve women in the N group and 11 in the Ob group were lost to follow-up, and there were 3 and 4 fetal losses in each group respectively. There were 228 live births in the N group and 238 in the Ob group. Preterm births (<37 weeks) were excluded from the analysis (respectively 6 in the N group and 12 in the Ob group), resulting in 222 N and 226 Ob mothers and their newborns in the final study group (Fig 1).

### Maternal and neonatal characteristics according to maternal BMI (Table 1)

Using the screening policy for gestational diabetes as described above, 45.2% of the Ob women were affected by gestational diabetes, half of which were diagnosed in the first trimester (49.5%, N = 49) and 36.4% (n = 36) in the second trimester. Among them, 52.1% required insulin. In the N group, 20% of the women (n = 41) were diagnosed with gestational diabetes, and 21.1% (n = 8) required insulin. In the N group, gestational hyperglycemia was diagnosed for most women at 32 weeks of gestation (58.5%, n = 24); it was considered and treated as gestational diabetes.

Birth weight was not statistically different between N and Ob groups (boys: 3456g±433 and 3392g±463, p = 0.24; girls: 3316g±402 and 3391g±408, p = 0.43, respectively). The proportion of large for gestational age or small for gestational was not different between the N and the Ob group (respectively 8.5% versus 10.7%, p = 0.5 and 9.5% versus 8.9%, p = 0.8). The sum of skinfolds was significantly higher in neonates in the Ob group (p<0.0001), as well as cord serum leptin (p<0.01) and C-peptide (p = 0.04) concentrations.

In a sex-specific analysis, sum of skinfolds, cord serum leptin concentrations and placental weight were significantly higher in girls in the Ob group compared to the N group (respectively, p<0.0001, p<0.01 and p<0.01), but not in boys (Table 1). C-peptide level was not different between the N and the Ob groups in boys (0.74 ng/mL±0.52 versus 0.83 ng/mL±0.49], p = 0.28) nor in girls (0.81 ng/mL±0.85] versus 1.07 ng/mL±0.84], p = 0.09).

### Maternal and neonatal characteristics according to BMI and diabetic status (Table 2)

A comparison was performed within the Ob group between non-diabetic obese women and diabetic obese women. Obese women with diabetes gained less weight during pregnancy and had lower serum leptin concentrations at delivery (p<0.05 for both criteria). The only significant difference between neonates in these two groups was that cord serum C-peptide concentrations were significantly higher in diabetic women (p<0.05).

A second analysis was performed to compare non-diabetic women in the N group and non-diabetic women in the Ob group. Maternal BMI, insulinemia, HOMA-IR at 37 weeks, HbA1c and leptin at delivery were significantly higher in non-diabetic obese women compared to non-diabetic normal women but gestational weight gain and adiponectin at delivery were lower in the Ob group (p<0.05 for all criteria). For the neonates, only skinfold thickness was significantly higher in the non-diabetic Ob group (p<0.05).

### Factors associated with neonatal anthropometrics, leptin and placental weight in multivariate analysis (Tables 3 and 4)

The association between maternal obesity and neonatal weight Z-score, sum of skinfolds and hormones was examined in a multivariate analysis. Only variables significant in the multivariate model are shown in Table 3.

**Table 3:** Factors associated with neonatal weight Z-score, sum of skinfolds, leptin and placenta weight in multivariate analysis. Data are presented with adjusted mean [standard error] for categorical variables or regression coefficient [standard error] for continuous variables.

	Adjusted mean [standard error]	Regression coefficient [standard error]	P-value
***Neonatal Weight Z-score N = 363***			
**Gestational weight gain**			0.02
More than recommended	0.02 [0.10]	-	
Less than recommended	-0.35 [0.11]	-	
Within recommended range	-0.21 [0.11]		
**Maternal smoking**			0.003
No	0.05 [0.05]	-	
Yes	-0.43 [0.15]	-	
**Parity**			<0.0001
Primigravida	-0.40 [0.10]	-	
Multigravida	0.02 [0.10]	-	
**HbA1c (%)**	-	0.36 [0.12]	0.003
***Neonatal sum of skinfolds (mm) N = 344***			
**Sex of the neonates and maternal obesity **			0.003*
Girls born to normal weight women	18 [0.6]	-	
Girls born to women with obesity	19.7 [0.5]	-	
Boys born to normal weight women	18.4 [0.6]	-	
Boys born to women with obesity	18.5 [0.5]	-	
**HbA1c (%)**	-	1.61 [0.4]	0.0003
**Hypertension**			0.04
No	19.6 [0.24]	-	
Yes	17.7 [0.88]	-	
**Center**			<0.0001
1	17.2 [0.5]	-	
2	20.1 [0.5]	-	
***Neonatal leptin (ng/mL) N = 323***			
**Sex of the neonates and maternal obesity**			<0.0001*
Girls born to normal weight women	11.3 [1.0]	-	
Girls born to women with obesity	15.3[1.0]	-	
Boys born to normal weight women	9.3 [1]	-	
Boys born to women with obesity	9 [1.0]	-	
**HbA1c (%)**	-	2.84 [1.23]	0.02
***Placental weight (g) N = 278***			
**Maternal Obesity**			0.006
18.5≤ BMI<25 kg/m^2^	560.4 [10.9]	-	
BMI≥30 kg/m^**2**^	602.4 [10.6]	-	

*p-value for interaction

**Table 4:** Multivariate analysis on skinfolds including HOMA-IR performed at 37 weeks in the model. The analysis was performed on data from 136 N and 124 Ob women. Data are presented with adjusted mean [standard error] for categorical variables or regression coefficient [standard error] for continuous variables. Only significant data are presented.

	Adjusted mean [standard error]	Regression coefficient [standard error]	P-value
*Neonatal sum of skinfolds (mm) N = 260*			
**Sex of the neonates and HOMA-IR**			<0.0001*
Girls		0.33 [0.08]	
Boys		0.17 [0.09]	
**HbA1c (%)**	-	1.92 [0.5]	<0.0001
**Smoking**			0.006
No	19.6 [0.3]	-	
Yes	17.8 [0.7]	-	
**Center**			<0.0001
1	17.3 [0.3]	-	
2	20.1 [0.6]	-	

*p-value for interaction

**Fig 2:**
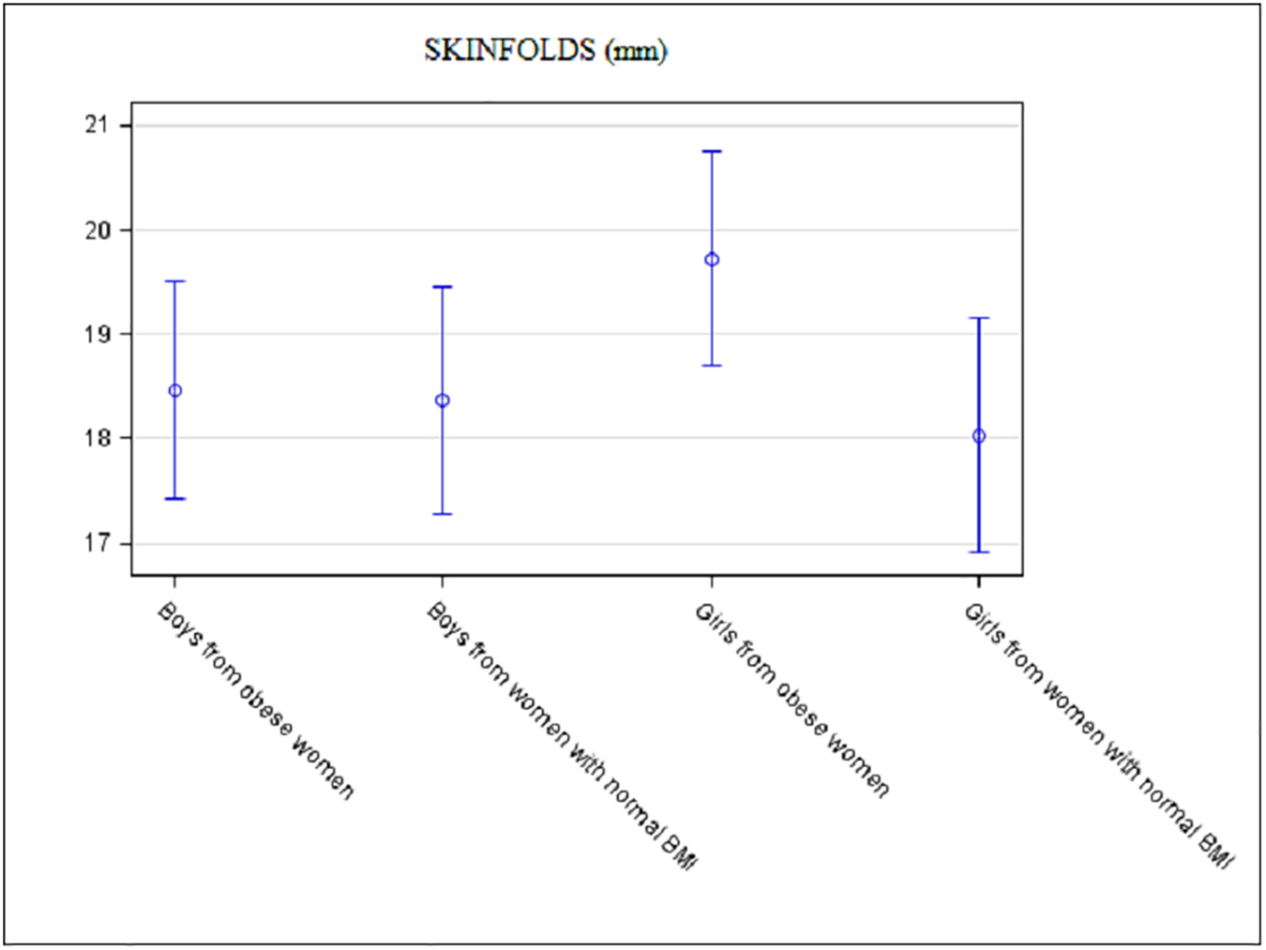
Adjusted neonatal sum of skinfolds according to maternal BMI and according to the sex of the neonate. Data are mean with 95% confidence limits.

**Fig 3:**
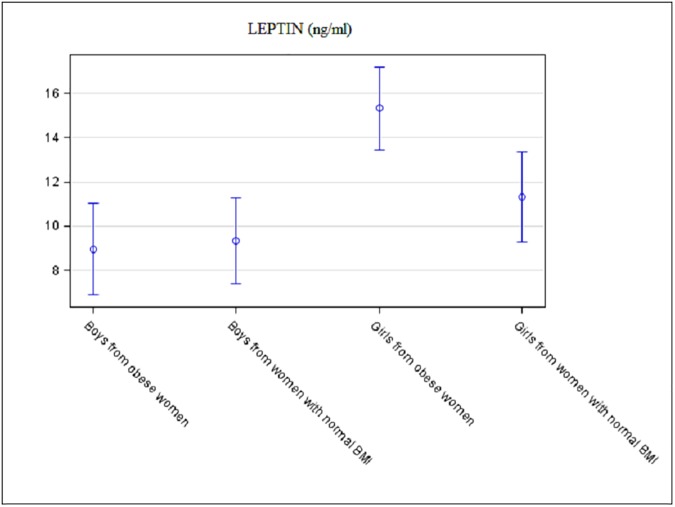
Adjusted neonatal cord leptin concentration according to maternal BMI and according to the sex of the neonate. Data are mean with 95% confidence limits.

There was no association between maternal obesity and weight Z-score of the neonate. The association between maternal obesity and newborn sum of skinfolds or cord leptin concentration differed according to infant sex (p = 0.003 and p**<**0.0001 for interaction, respectively). Sum of skinfolds and cord leptin concentration were significantly increased in the girls in the Ob group (18.0mm±0.6 versus 19.7mm±0.5, p = 0.004 and 11.3ng/mL±1.0 versus 15.3ng/mL±1.0, p = 0.02, respectively). For boys, there was no difference between groups for sum of skinfolds and cord leptin concentration: 18.4mm±0.6 versus 18.5mm±0.5, p = 0.9 and 9.3ng/mL±1.0 versus 9.0ng/mL±1.0, p = 0.9, respectively (Fig 2 and Fig 3).

Maternal diabetes was not significantly associated with neonatal weight Z-score, sum of skinfolds, nor leptin levels.

Regardless of diabetes status, maternal HbA1c at delivery was associated with weight Z-score, skinfold and leptin concentrations. A 1-point increase in HbA1c corresponded respectively to a 0.36 increase in weight Z-score (p = 0.003), a 1.61 mm increase in sum of skinfolds (p = 0.0003) and a 2.84 ng/mL increase in cord leptin concentration (p = 0.02).

In the multivariate analysis performed on 143 women in the Ob group and 135 women in the N group, placental weight was significantly higher in the Ob group (602.4g±10.6 versus 560.4±10.9, p = 0.006), but there was no association between placental weight and maternal diabetic status.

Despite 37% missing data for the HOMA-IR at 37 weeks, an additional multivariate analysis based on data from the 136 N and 124 Ob women where this information was available was performed (Table 4). When HOMA-IR at 37 weeks was added to the full model for skinfold, there was no association between sum of skinfolds and HOMA-IR in boys (p = 0.07), but a strong association was observed in girls (p<0.0001). A 1-point increase in HOMA-IR corresponded to 0.33mm increase in the sum of skinfolds in girls.

## Discussion

This study shows that regardless of gestational diabetes, maternal obesity was not associated with increased birthweight but it was nevertheless associated with higher fat mass and leptin in girls, but not in boys. A strong association between maternal insulin resistance and fat mass in girls was also found.

We designed this study to discriminate the effect of maternal obesity and that of treated gestational diabetes on birthweight and neonatal fat mass. A recent meta-analysis had concluded that maternal obesity increases by about two folds the risk for a neonate of being large for gestational age (>90^th^ percentile) [9]. The authors emphasized, however, that factors other than maternal BMI, such as gestational diabetes, may have contributed to increasing birthweight.

Although our current practice is to screen for diabetes only in the first and/or the second trimester, in this study, women were also systematically screened for gestational diabetes in the third trimester, regardless of maternal BMI. Based on the fact that insulin resistance increases throughout pregnancy, our hypothesis was that adding a third trimester OGTT would enable the detection of gestational diabetes that would have been missed by second trimester screening alone. Thus, maternal glycemic control would be optimized, limiting the effect of hyperglycemia on birthweight and fat mass. Here, applying the IADPSG criteria for diagnosis of gestational diabetes, the rate of gestational diabetes was particularly high compared to the 17.8% (range: 9.3–25.5%) observed in the HAPO study population as a whole, regardless of maternal BMI [10]. Nevertheless, in the N group, the 20% rate of gestational diabetes was mainly due to the cases diagnosed in the third trimester. The rate of gestational diabetes diagnosed in the first and the second trimesters was only 7.66% (17/222), in agreement with the prevalence of gestational diabetes recently reported in the French population [11]. The high incidence of diabetes diagnosed in the first and the second trimester in the Ob group (37.6%, 85/226) could be linked to the ethnic diversity of this group that may also influence the frequency of diabetes. Indeed, a frequency of 31.5% of gestational diabetes using the IADPSG criteria was previously reported in a multi-ethnic population [12]. Also, as most of the obese women were diagnosed during the first trimester, it can not be excluded that a percentage of women had hyperglycemia prior to pregnancy. The rate of hyperglycemia detected in the third trimester is mainly due to women in the N group compared to the Ob group (58.5% versus 14.1%). These data illustrate that in normal weight women, insulin resistance increases throughout pregnancy and is highest in the third trimester in contrast to what is observed in many obese women who already have higher insulin resistance in early gestation [13]. In the context of screening and treatment of diabetes throughout pregnancy, maternal obesity was not associated with excess birthweight, but was associated with higher fat mass deposition in girls. The same association was observed between maternal obesity and cord leptin levels in girls. Fetal leptin level is believed to be independent from maternal and placental contributions and to correlate with fetal fat mass [14].

The comparison between non diabetic women and their neonates in the N and the Ob groups showed that birthweight was not different but that sum of skinfolds was significantly higher in the Ob group. These results are similar to those reported a few years ago by Sewel et al. who observed, in women with normal second trimester OGTT, a moderate difference in birthweight and a significant increase in neonatal fat mass in the group with BMI≥25 kg/m^2^ (n = 76) compared to the group with BMI<25 kg/m^2^ (n = 144). There was, however, no association between fat mass and fetal sex in the group with BMI≥25 kg/m^2^ [8].

Different factors of maternal metabolism may contribute to higher fat mass in neonates born to women with obesity. In the present study, the comparison between non-diabetic and diabetic obese women showed that they had the same level of insulin resistance as measured by HOMA-IR at 37 weeks and the same level of HbA1c at delivery. Furthermore, as shown elsewhere [15], we observed that pregnant women with obesity who have normal glucose tolerance still had a higher glucose profile (glycemia and HbA1c) than pregnant women of normal weight, thereby exposing the fetus to relative hyperglycemia. This means that regardless of gestational diabetes, deregulation of glucose metabolism is present in obese women and may contribute to fat mass in the neonates.

Furthermore, it was showed that maternal insulin resistance that is associated with higher maternal circulating free fatty acid and triglyceride concentrations, contributes to neonatal adiposity [16]. Here, Ob women had greater insulin resistance in the last month of pregnancy than N women. The multivariate analysis showed that maternal HOMA-IR had a strong effect on skinfold value in girls. Recently published studies conducted in unselected pregnant women identified a positive correlation between neonatal and maternal fat mass and HOMA-IR only in girls [17, 18]. These results are in agreement with our findings in a selected group of women with obesity.

We found a significant higher placental weight in girls in the Ob Group, compared to the N group but there was no difference in placental weight for boys. Nevertheless, in the multivariate analysis, placental weight was only related to maternal obesity without sex interaction, maybe because of many missing data in this analysis. Catalano et al. speculated that decreased pregravid insulin sensitivity in women with obesity may lead to abnormal early placental development which later affects placental transport of nutrients, especially lipid transport [13]. Recently, different studies in women with high BMI showed that placental adaptations, including placental biometry or histopathology, depend on fetal sex, with significant changes occurring only in girls [19, 20]. There is also evidence from human and animal studies that differential molecular expression according to the sex of the placenta may lead to different nutritional conditions for the fetus [21]. Brass et al. showed that placental uptake of oleic acid was suppressed and that the expression of the placental transporter CD36 was lower in male newborn of women with obesity, but not in female newborns [22]. In animal models, sexual dimorphism was observed in the placental phenotype in response to high fat and control maternal diets in rabbits [23]. Moreover, in mice fed a high fat diet, male and female placenta diverged in epigenetic and transcriptomic analyses related to diet response [24].

Being the active interface between the mother and the fetus, the placenta appears to be a relevant target to better understand the molecular link between maternal obesity, insulin-resistance and fetal growth.

Limitations of this study include the fact that cord C-peptide and glycemia measurements were not available in 40% of the neonates, and placental weight and HOMA-IR were not available in 37% of the mothers. Skinfold thickness is an indirect measurement of adiposity, but it correlates in neonates with fat mass values determined by dual-energy X-ray absorptiometry (DXA), a validated method for determining body fat [7]. Although pediatricians in both centers underwent rigorous training for the measurement of skinfolds, sum of skinfolds was significantly different between the two centers. This parameter was included in the multivariate analysis and in spite of the difference in measurement, there was a strong correlation between maternal obesity and skinfold in girls. Despite the fact that women with known type 1 or type 2 diabetes were excluded, as discussed above, it cannot be ruled out that some women had hyperglycemia prior to becoming pregnant. Nevertheless, this would not have changed the results. There was also a notable difference in ethnicity among the different groups, but this was taking into account in the statistical model.

Finally, we cannot formally exclude that women included in the study have changed their lifestyle because of enrolment in the study. In such a case, these changes in their lifestyle, particularly in their diet, would have been probably minor. Obese women had taken on average less weight than lean women during pregnancy, but most of them had taken more weight anyway than recommended by the IOM. Furthermore, the metabolic characteristics of the obese women we observed were very different from those of non-obese women. Therefore, potential changes in lifestyle had little effect on the maternal environment to which the fetus was exposed.

## Conclusion

Regardless of gestational diabetes, maternal obesity is not associated with increased birth weight but is associated with increased neonatal adiposity in girls only.

A better understanding of how boys and girls differentially adapt to intrauterine environmental disturbances should help to improve our knowledge of fetal programming and to anticipate future disease susceptibility.

## Supporting information

S1 File Trial study protocol in English. (DOC)

S2 File Trial study protocol in French. (DOC)

S3 File CONSORT checklist. (PDF)
