# Supplementary material for: Effect of maternal obesity on birthweight and neonatal fat mass: A prospective clinical trial
Source: PLoS One. 2017 Jul 27;12(7):e0181307. doi: 10.1371/journal.pone.0181307 (PMC5531500; doi:10.1371/journal.pone.0181307)
Supplement: S1 File — (DOC) [file pone.0181307.s001.doc]

# PHRC 2009 - REGIONAL CALL FOR PROPOSALS

# APPLICATION

**STUDY TITLE:**

**Impact of maternal obesity on fetal development and perinatal events**

**Analysis of the determinants of fetal growth.**

**Principal investigator:**  Pr MITANCHEZ

Department of Neonatology

Hôpital Armand Trousseau

26 avenue du Dr Arnold Netter

7501é PARIS FRANCE

Tél : + 33 1 44 73 61 91 /Fax : + 33 1 44 73 68 92

Email: delphine.mitanchez[@trs.aphp.fr](mailto:jean-philippe.collet@psl.aphp.fr)

Sponsor: Assistance Publique - Hôpitaux de Paris

DRCD – Hôpital St Louis

Project director:

Assistant project director:

**URC:** URC Pitié-Salpêtrière – Charles-Foix

Pr A. Mallet

GH Pitié-Salpêtrière

47, Boulevard de l’hôpital

75013 PARIS France

Tél : + 33 1 42 16 16 53

Email: [urc.pslcfx@psl.aphp.fr](mailto:urc.pslcfx@psl.aphp.fr)

**TABLE OF CONTENTS**

[**1. General information:** 4](#__RefHeading___Toc475518719)

[**1.1: Complete study title:** 4](#__RefHeading___Toc475518720)

[**1.2: Name and address of sponsor:** 4](#__RefHeading___Toc475518721)

[**1.3: Names and title of persons authorized to sign the protocol and any modifications in the sponsor's name:** 4](#__RefHeading___Toc475518722)

[**1.4: Name, title, and address of individual responsible for the study for the sponsor:** 4](#__RefHeading___Toc475518723)

[**1.5: Names and titles of investigators:** 4](#__RefHeading___Toc475518724)

[**2. Scientific justification and general description of the research:** 5](#__RefHeading___Toc475518725)

[**2.1 Summary of findings from nonclinical studies and from clinical trials available and relevant to this trial:** 5](#__RefHeading___Toc475518726)

[**2.2** **Summary of benefits, where appropriate, and predictable or known risks for the subjects participating in this study:** 7](#__RefHeading___Toc475518727)

[**2.3** **Description of the study population:** 8](#__RefHeading___Toc475518728)

[**2.4 References to the scientific literature:** 8](#__RefHeading___Toc475518729)

[**3. Study objectives:** 11](#__RefHeading___Toc475518730)

[**4. Study design:** 11](#__RefHeading___Toc475518731)

[**4.1 Principal and secondary outcome measures:** 11](#__RefHeading___Toc475518732)

[**4.2 Study methodology and feasibility:** 12](#__RefHeading___Toc475518733)

[**4.3 Steps taken to reduce and avoid bias:** 12](#__RefHeading___Toc475518734)

[**4.4 Precise description of the study process** 13](#__RefHeading___Toc475518735)

[**4.5 Planned duration of individual participation and description of the trial calendar:** 15](#__RefHeading___Toc475518736)

[**4.6 Description of rules for temporary or permanent stopping:** 17](#__RefHeading___Toc475518737)

[**4.7 Data to be collected directly in the case report forms:** 17](#__RefHeading___Toc475518738)

[**5. Selection and exclusion of study subjects**: 17](#__RefHeading___Toc475518739)

[**5.1 Inclusion criteria for subjects agreeing to participate in this study:** 17](#__RefHeading___Toc475518740)

[**5.2 Criteria for exclusion of study subjects:** 17](#__RefHeading___Toc475518741)

[**5.3 Procedure for early withdrawal from the study or for exclusion of a study subject, and procedure for their follow-up:** 18](#__RefHeading___Toc475518742)

[**6. Treatment administered to subjects agreeing to participate in this study:** 18](#__RefHeading___Toc475518743)

[**7. Efficacy assessment:** 18](#__RefHeading___Toc475518744)

[**8. Safety assessment:** 18](#__RefHeading___Toc475518745)

[**9. Adverse effects:** 18](#__RefHeading___Toc475518746)

[**10. Statistics and data management:** 18](#__RefHeading___Toc475518747)

[**10.1 Description of the statistical methods to be used, including the timing of any planned interim analyses.** 18](#__RefHeading___Toc475518748)

[• *Principal statistical analyses* 18](#__RefHeading___Toc475518749)

[• *Secondary analyses* 19](#__RefHeading___Toc475518750)

[• *Calendar of analyses* 19](#__RefHeading___Toc475518751)

[**10.2 Planned number of study subjects, and planned number of subjects at each study center, with its statistical justification.** 19](#__RefHeading___Toc475518752)

[**10.3 Αlpha (type 1) risk** 19](#__RefHeading___Toc475518753)

[**10.4 Statistical criteria for halting the study** 19](#__RefHeading___Toc475518754)

[**10.5 Method for taking missing, unused, or invalid data into account** . 19](#__RefHeading___Toc475518755)

[**10.6 Management of modifications to the analysis plan of the initial strategy.** 19](#__RefHeading___Toc475518756)

[**10.7 Selection of subjects to be included in the analyses** 20](#__RefHeading___Toc475518757)

[**10.8 Data management** 20](#__RefHeading___Toc475518758)

[**10.9 Supervisor of data analysis and software** 20](#__RefHeading___Toc475518759)

[**11. Budget justification:** 20](#__RefHeading___Toc475518760)

[**12. Right of access to data and source documents** 20](#__RefHeading___Toc475518761)

[**13. Quality control and assurance** 21](#__RefHeading___Toc475518762)

[**14. Legal and ethical considerations** 21](#__RefHeading___Toc475518763)

[**14.1 Request for authorization from AFSSAPS.** 21](#__RefHeading___Toc475518764)

[**14.2 Request for an opinion from** the Patient Protection Committee 21](#__RefHeading___Toc475518765)

[**14.3 Modifications** 21](#__RefHeading___Toc475518766)

[**14.4 CNIL Declaration** 21](#__RefHeading___Toc475518767)

[**14.5 Information note and Informed consent** 21](#__RefHeading___Toc475518768)

[**15. Data treatment and storage of documents and data related to the research 21**](#__RefHeading___Toc475518769)

[**16. Funding and insurance.** 22](#__RefHeading___Toc475518770)

[**17. Rules relative to publication** 22](#__RefHeading___Toc475518771)

[**18. List of appendices** 22](#__RefHeading___Toc475518772)

**RESEARCH PROTOCOL**

**1. General information:**

**1.1: Complete study title:**

**Impact of maternal obesity on fetal development and perinatal events**

**Analysis of the determinants of fetal growth.**

**- Short title: Obese mothers' newborns**

**- Sponsor's protocol code number:**

**- Version number:**

**- Date of protocol:** 26/11/2008

**1.2: Name and address of sponsor:**

Assistance Publique - Hôpitaux de Paris

DRCD – Hôpital St Louis

**- Legal representatives in the European Community:**

**- Organization responsible for monitoring the trial**

**1.3: Names and title of persons authorized to sign the protocol and any modifications in the sponsor's name:**

**1.4: Name, title, and address of individual responsible for the study for the sponsor:**

**1.5: Names and titles of investigators:**

Principal Investigator: Pr MITANCHEZ Delphine, University professor-hospital physician, Head of Neonatology department, Armand Trousseau and Pitié Salpêtrière Hospitals, GHU-East.

Names and titles of others collaborating on this study: M Dommergues (PU-PH),

J Nizard (PHU), C Renaud (PH), I Demontgolfier (PH), A Wallet (PH), M Moreno (PHC), JM Jouannic (PU-PH), C De Carne (CCA), A Basdevant (PU-PH), C Ciangura (CCA), S Jaqueminet (PH), A Grimaldi (PU-PH), C Sachon, (PH), JM Lacorte (MCU-PH), C Coussieu (MCU-PH), V Frochot (AHU), MA Charles (ATT, DR).

**- Site of study:**

Maternity unit, Hôpital Pitié-Salpêtrière. 47, boulevard de l’hôpital. 75013 PARIS.

Perinatal Center, Hôpital Armand Trousseau 26 avenue du Dr Arnold Netter, 75012 PARIS

**2. Scientific justification and general description of the research:**

**2.1 Summary of findings from nonclinical studies and from clinical trials available and relevant to this trial:**

The proportion of women who are overweight at the beginning of their pregnancy has been increasing in recent decades. In France, the national epidemiologic survey on obesity and overweight published in September 2006 (ObEpi survey) confirmed the previously observed increase in the incidence of obesity [1]. Nearly 14 million French people are overweight or obese. This rate is increasing faster and more strongly among women than men (+64% versus +40%): 23% of women are overweight and 13% obese. These women are exposed to substantial morbidity; for those of child-bearing age, it includes complications during pregnancy.

The two most frequent complications of obesity during pregnancy are chronic hypertension with the risk of preeclampsia and gestational diabetes. Maternal obesity is also associated with a higher frequency of cesarean deliveries, fetal and neonatal deaths and malformations, and of very preterm births, as well as with a high rate of complications in the newborn, related in part to macrosomia [2-6].

The neonatal particularities of infants born to obese mothers are not well known, partly because the data from the literature comes mostly from obstetric studies that provide only summary information about immediate neonatal morbidity, based on data collected in the delivery room. No available study on this subject has been conducted by a neonatology team focusing on the first days of life. At first look, the fetal and neonatal complications described in pregnancies of obese women appear analogous to those described for the children of women with diabetes during pregnancy, and the current data do not allow the determination that any specific disorder is related to maternal obesity, independently of diabetes. Nonetheless, it appears that the modifications to the intrauterine environment secondary to maternal obesity could by themselves lead to specific fetal and neonatal consequences, as shown by data about malformations. The risk of fetal malformations appears higher among obese, compared to non-obese, pregnant women; these concern primarily neural tube defects but can also affect other organs, including the heart, the gastrointestinal tract, and the lungs [7-11]. Some attribute these malformations to the frequent association between obesity and diabetes, but other studies have shown that obesity is an independent risk factor for fetal malformations [12]. Accordingly, an association with the risks of congenital diaphragmatic hernia and of omphalocele was found, neither generally reported in diabetes [8].

Some studies have shown that obese mothers' newborns have a higher birthweight and that this is related to the mother's preconception BMI (body mass index) and to her weight gain during pregnancy, independently of diabetes [13, 14]. The higher birthweight in the newborns of obese women without diabetes may be associated only with an increase in fat mass, but not lean body mass [15]. The body composition of newborns can be assessed from measurements that allow a reliable estimate of the lean and fat body mass [16]. These measurements include weight and height, from which BMI (weight/height^2^) and the ponderal index (weight/height^3^) can be calculated, as well as head circumference and the following skinfold thicknesses: tricipital, bicipital, supra-iliac, and subscapular. Estimates of the fat mass by measurement of these skinfolds and by absorptiometry correlate well [17].

The mechanisms causing excess fetal growth during the pregnancies of obese women are not known. We can assume that these modifications result from changes in the maternal metabolism. Accordingly, several authors have shown the influence of the quality of maternal nutritional intake, in particular macronutrients (proteins, carbohydrates and lipids), on fetal growth [18, 19]. A recent study points out the association between maternal nutritional intake during pregnancy and changes in the production of hormones regulating metabolism, such as leptin, adiponectin, and resistin. Some of these hormones stimulate the placental transportation of nutrients [20]. There may thus be an association between overweight and an increase in maternal metabolism hormones that could induce excess fetal growth by increasing placental transportation of nutrients [21].

We now know several fetal factors that play a role in fetal growth, but we do not yet know how maternal obesity might influence their expression.

Insulin is an essential growth factor for the fetus. The excessive growth of infants born to diabetic mothers is therefore attributed to fetal hyperinsulinism secondary to maternal hyperglycemia. Several mechanisms produce the trophic effect of insulin: insulin stimulates the entry and utilization of nutrients by insulin-sensitive tissues (including adipose tissue); it has a direct mitogenic effect; and finally, it interacts with the IGF (Insulin-like Growth Factor) system to stimulate production of IGF-1 [22]. The IGFs play a key role in regulating fetal and postnatal growth. IGF-2 is involved principally in fetal growth, while its homolog, IGF-1, is involved during both the fetal and postnatal periods. These two growth factors act on the same receptor tyrosine kinase, IGF-R, and are essential mediators of the growth hormone. IGF-1 production is lower in infants with growth restriction and higher in newborns with macrosomia born to mothers with diabetes. IGF-1 shares structural homology with proinsulin and possesses metabolic effects analogous to those of insulin [23].

Leptin is a protein produced mainly by adipose tissue but also by the placenta. Fetal adipose tissue is a major source of leptin, the production of which follows the development of this tissue: the leptin level is low during the first half of pregnancy and then rises strongly during the third trimester. Its production may be regulated by insulin, but also by fetal hypoxia [24]. Maternal leptin does not cross the placenta. The vast majority of placental leptin is released into the maternal circulation, with only a tiny fraction entering the fetus [25]. Some authors consider that the leptin level at birth simply reflects the newborn's body fat mass [26]. Others, however, consider that it is an important factor in fetal growth and development and that its action is independent of that of insulin and of IGF-1 [27-29]. Its mechanism of action on fetal growth nonetheless remains unknown.

Adiponectin is another protein secreted exclusively by adipose tissue. It is paradoxically lower in obese individuals and increases during weight loss. Adiponectin is an important modulator of insulin action and of glucose metabolism — both elements that regulate fetal growth. It is accordingly a possible factor in regulating this growth. Different studies have analyzed the profile of adiponectin expression in newborns as a potential function of in utero growth. The results are often contradictory, but the profile of this expression differs from that observed in adults. That is, levels in newborns are higher than those in adults [30, 31]. Birthweight is thought to be positively correlated with the adiponectin concentrations assayed in the cord blood of preterm infants, growth-restricted newborns, and normal-weight newborns born at term [32]. On the other hand, this concentration may be lower in newborns with macrosomia [33]. Some consider that adiponectin levels are unrelated to fat mass in fetuses, although they are related in adults [34]. Finally, a recent study shows that the umbilical cord adiponectin level is correlated only with height at birth [35]. This hormone may thus act by increasing fetal insulin sensitivity and thereby potentiating the effect of IGF-1. Adiponectin has never been studied specifically in the newborns of obese mothers. Moreover, it has several isoforms (globule-shaped, hexameric forms, and high molecular-weight forms) [36] and their variations may have different metabolic significance [37].

These different growth factors also play a role in glucose homeostasis. Hypoglycemia in neonates born to diabetic mothers has been widely studied and is attributed to hyperinsulinism that persists after birth. Nonetheless, a relation between the risk of hypoglycemia and the extent of macrosomia is not always observed [38]. A single study has reported a higher risk of hypoglycemia in the newborn infants of obese mothers [39]; a positive correlation has also been observed between maternal BMI at the beginning of pregnancy and umbilical cord levels of insulin and C-peptide [38].

We propose to study the newborn infants of obese women, that is, women with a preconception BMI ≥ 30 kg/m^2^; this criterion identifies a population of high-weight women not sufficiently accessible in general population-based studies. We start with the hypothesis that these newborns have a higher mean birthweight than those of normal-weight mothers. We want to assess the consequences of maternal obesity:

- on the newborn's weight and fat mass,
- on the functioning of the fat mass, specifically on its production of leptin and adiponectin,
- on levels of the fetal growth factors (insulin, IGF-1)
- and on the newborn's glucose metabolism.

We will determine the role of obesity and diabetes on each of these elements.

**2.2** **Summary of benefits, where appropriate, and predictable or known risks for the subjects participating in this study:**

This study comports no risk for either mother or child, and its principal objective provides no immediate benefits.

Obstetric management of the pregnancy will be according to the usual standard of care. There will be only one supplementary test, to screen for diabetes by an oral glucose tolerance test, during the third trimester; in addition, two blood samples will be taken at the same time as other samples or when starting an infusion. Their volume will be small (2 mL for the first sample, and 12 mL for the second).

The study will not modify the standard management of the newborn at all. The newborns’ blood samples for the study will come from umbilical cord blood.

The data will be used anonymously.

**2.3** **Description of the study population:**

The study population will be singleton newborn infants of obese mothers (prepregnancy BMI ≥ 30 kg/m^2^) receiving prenatal care and giving birth in the two study maternity units: at Pitié Salpêtrière Hospital and at Trousseau Hospital, both in Paris. The control population comprises singleton newborn infants of non-obese mothers (18.5 ≤BMI< 25 kg/m^2^ before pregnancy) with prenatal care and delivery at one of these two maternity units.

**2.4 References to the scientific literature:**

1. Charles MA, Eschwege E, Basdevant A. Monitoring the obesity epidemic in france: the obepi surveys 1997-2006. Obesity (Silver Spring) 2008;16(9):2182-6.

2. Callaway LK, Prins JB, Chang AM, McIntyre HD. The prevalence and impact of overweight and obesity in an Australian obstetric population. Med J Aust 2006;184(2):56-9.

3. Cedergren MI. Maternal morbid obesity and the risk of adverse pregnancy outcome. Obstet Gynecol 2004;103(2):219-24.

4. Weiss JL, Malone FD, Emig D, Ball RH, Nyberg DA, Comstock CH, et al. Obesity, obstetric complications and cesarean delivery rate--a population-based screening study. Am J Obstet Gynecol 2004;190(4):1091-7.

5. Kristensen J, Vestergaard M, Wisborg K, Kesmodel U, Secher NJ. Pre-pregnancy weight and the risk of stillbirth and neonatal death. Bjog 2005;112(4):403-8.

6. Cnattingius S, Bergstrom R, Lipworth L, Kramer MS. Prepregnancy weight and the risk of adverse pregnancy outcomes. N Engl J Med 1998;338(3):147-52.

7. Ray JG, Wyatt PR, Vermeulen MJ, Meier C, Cole DE. Greater maternal weight and the ongoing risk of neural tube defects after folic acid flour fortification. Obstet Gynecol 2005;105(2):261-5.

8. Waller DK, Shaw GM, Rasmussen SA, Hobbs CA, Canfield MA, Siega-Riz AM, et al. Prepregnancy obesity as a risk factor for structural birth defects. Arch Pediatr Adolesc Med 2007;161(8):745-50.

9. Watkins ML, Rasmussen SA, Honein MA, Botto LD, Moore CA. Maternal obesity and risk for birth defects. Pediatrics 2003;111(5 Part 2):1152-8.

10. Cedergren M, Kallen B. Maternal obesity and the risk for orofacial clefts in the offspring. Cleft Palate Craniofac J 2005;42(4):367-71.

11. Cedergren MI, Kallen BA. Maternal obesity and infant heart defects. Obes Res 2003;11(9):1065-71.

12. Hendricks KA, Nuno OM, Suarez L, Larsen R. Effects of hyperinsulinemia and obesity on risk of neural tube defects among Mexican Americans. Epidemiology 2001;12(6):630-5.

13. Ehrenberg HM, Mercer BM, Catalano PM. The influence of obesity and diabetes on the prevalence of macrosomia. Am J Obstet Gynecol 2004;191(3):964-8.

14. Okun N, Verma A, Mitchell BF, Flowerdew G. Relative importance of maternal constitutional factors and glucose intolerance of pregnancy in the development of newborn macrosomia. J Matern Fetal Med 1997;6(5):285-90.

15. Sewell MF, Huston-Presley L, Super DM, Catalano P. Increased neonatal fat mass, not lean body mass, is associated with maternal obesity. Am J Obstet Gynecol 2006;195(4):1100-3.

16. Koo WW, Walters JC, Hockman EM. Body composition in neonates: relationship between measured and derived anthropometry with dual-energy X-ray absorptiometry measurements. Pediatr Res 2004;56(5):694-700.

17. Schmelzle HR, Fusch C. Body fat in neonates and young infants: validation of skinfold thickness versus dual-energy X-ray absorptiometry. Am J Clin Nutr 2002;76(5):1096-100.

18. Godfrey K, Robinson S, Barker DJ, Osmond C, Cox V. Maternal nutrition in early and late pregnancy in relation to placental and fetal growth. Bmj 1996;312(7028):410-4.

19. Moore VM, Davies MJ, Willson KJ, Worsley A, Robinson JS. Dietary composition of pregnant women is related to size of the baby at birth. J Nutr 2004;134(7):1820-6.

20. Jones HN, Powell TL, Jansson T. Regulation of placental nutrient transport--a review. Placenta 2007;28(8-9):763-74.

21. Jansson N, Nilsfelt A, Gellerstedt M, Wennergren M, Rossander-Hulthen L, Powell TL, et al. Maternal hormones linking maternal body mass index and dietary intake to birth weight. Am J Clin Nutr 2008;87(6):1743-9.

22. Hill DJ, Petrik J, Arany E. Growth factors and the regulation of fetal growth. Diabetes Care 1998;21 Suppl 2:B60-9.

23. Holt RI, Simpson HL, Sonksen PH. The role of the growth hormone-insulin-like growth factor axis in glucose homeostasis. Diabet Med 2003;20(1):3-15.

24. Hytinantti TK, Koistinen HA, Teramo K, Karonen SL, Koivisto VA, Andersson S. Increased fetal leptin in type I diabetes mellitus pregnancies complicated by chronic hypoxia. Diabetologia 2000;43(6):709-13.

25. Alexe DM, Syridou G, Petridou ET. Determinants of early life leptin levels and later life degenerative outcomes. Clin Med Res 2006;4(4):326-35.

26. Hauguel-de Mouzon S, Lepercq J, Catalano P. The known and unknown of leptin in pregnancy. Am J Obstet Gynecol 2006;194(6):1537-45.

27. Hassink SG, de Lancey E, Sheslow DV, Smith-Kirwin SM, O'Connor DM, Considine RV, et al. Placental leptin: an important new growth factor in intrauterine and neonatal development? Pediatrics 1997;100(1):E1.

28. Wiznitzer A, Furman B, Zuili I, Shany S, Reece EA, Mazor M. Cord leptin level and fetal macrosomia. Obstet Gynecol 2000;96(5 Pt 1):707-13.

29. Vatten LJ, Nilsen ST, Odegard RA, Romundstad PR, Austgulen R. Insulin-like growth factor I and leptin in umbilical cord plasma and infant birth size at term. Pediatrics 2002;109(6):1131-5.

30. Kamoda T, Saitoh H, Saito M, Sugiura M, Matsui A. Serum adiponectin concentrations in newborn infants in early postnatal life. Pediatr Res 2004;56(5):690-3.

31. Pardo IM, Geloneze B, Tambascia MA, Barros-Filho AA. Hyperadiponectinemia in newborns: relationship with leptin levels and birth weight. Obes Res 2004;12(3):521-4.

32. Tsai PJ, Yu CH, Hsu SP, Lee YH, Chiou CH, Hsu YW, et al. Cord plasma concentrations of adiponectin and leptin in healthy term neonates: positive correlation with birthweight and neonatal adiposity. Clin Endocrinol (Oxf) 2004;61(1):88-93.

33. Mazaki-Tovi S, Kanety H, Pariente C, Hemi R, Schiff E, Sivan E. Cord blood adiponectin in large-for-gestational age newborns. Am J Obstet Gynecol 2005;193(3 Pt 2):1238-42.

34. Lindsay RS, Walker JD, Havel PJ, Hamilton BA, Calder AA, Johnstone FD. Adiponectin is present in cord blood but is unrelated to birth weight. Diabetes Care 2003;26(8):2244-9.

35. Inami I, Okada T, Fujita H, Makimoto M, Hosono S, Minato M, et al. Impact of serum adiponectin concentration on birth size and early postnatal growth. Pediatr Res 2007;61(5 Pt 1):604-6.

36. Kadowaki T, Yamauchi T, Kubota N, Hara K, Ueki K, Tobe K. Adiponectin and adiponectin receptors in insulin resistance, diabetes, and the metabolic syndrome. J Clin Invest 2006;116(7):1784-92.

37. Basu R, Pajvani UB, Rizza RA, Scherer PE. Selective downregulation of the high molecular weight form of adiponectin in hyperinsulinemia and in type 2 diabetes: differential regulation from nondiabetic subjects. Diabetes 2007;56(8):2174-7.

38. Soltani KH, Bruce C, Fraser RB. Observational study of maternal anthropometry and fetal insulin. Arch Dis Child Fetal Neonatal Ed 1999;81(2):F122-4.

39. Doherty DA, Magann EF, Francis J, Morrison JC, Newnham JP. Pre-pregnancy body mass index and pregnancy outcomes. Int J Gynaecol Obstet 2006;95(3):242-7.

**3. Study objectives:**

**The principal objective** of this project is to study whether the infants of obese mothers have differences at birth in body composition, assessed by weight and fat mass, compared with the infants of normal-weight mothers. We will focus especially on differentiating the role of maternal obesity from that of her diabetes.

**The secondary objectives** are to study:

- the differences in the functioning of adipose tissue of the newborn infants of obese, compared with non-obese, mothers, assessed by leptin and adiponectin assays;
- the relations between the different growth factors, such as IGF-1 and insulin, and the infant's birth weight and fat mass (because hemolysis is frequent in cord blood samples and insulin cannot be assayed in these conditions, insulin will be assessed by an assay of C-peptide, since they are present in equimolar quantities);
- the relations between the maternal metabolic indicators (leptin, adiponectin, glycosylated hemoglobin (HbA1c), fructosamine and blood glucose), the newborn's fat mass (quantity and functioning) and growth factors (C-peptide and IGF-1);
- the relations between the course of newborn's blood glucose level in the immediate postnatal period and the hormone concentrations measured in the umbilical cord blood (C-peptide, IGF-1, leptin, and adiponectin);
- estimation of the rates of perinatal mortality, morbidity, and hypoglycemia.

**4. Study design:**

**4.1 Principal and secondary outcome measures:**

The principal **outcome measures** are:

- The physical measurements at birth of the infants of obese mothers, that is, the ponderal index, sum of skinfold thickness measurements (skinfolds), and weight. These anthropometric indicators make possible a reliable estimate of the newborn's adiposity (fat mass).

The **secondary outcome measures** are:

- The levels of hormones involved in fetal growth regulation or reflecting the functioning of the fetal fat mass, measured in cord blood: C-peptide, IGF-1, leptin, adiponectin, and its different isoforms.

- The maternal metabolic indicators:

- the existence (or not) of diabetes and the modalities of its treatment
- fructosamine and HbA1c assays at delivery (reflecting mean maternal glycemic control during the weeks preceding the assay)
- maternal leptin and adiponectin levels at delivery, to characterize the maternal adipose tissue.

- Glycemic control in the newborn during the first days of life, assessed by monitoring capillary blood glucose.

-Perinatal events: mortality, malformations, obstetric trauma, perinatal asphyxia, respiratory distress, preterm delivery, and hypoglycemia.

**4.2 Study methodology and feasibility:**

This will be a two-center prospective observational study comparing exposed (infants of obese mothers) and non-exposed (infants of non-obese mothers) newborns.

**Chronological study diagram**

Birth

=

D0

Neonatal period

Pregnancy

1st visit

2^nd^ visit

Consent; fasting blood glucose

oral glucose tolerance test 75 g

24-28 weeks

1 visit/month

Weight gain, blood pressure proteinuria

Oral glucose tolerance test 75 g

Ultrasound

FHR

± management by diabetes specialists

Maternal sample: HbA1c

fructosamine, leptin, adiponectin

Samples from

cord blood

Apgar, pH

Weight, height, head circumference

Skinfold measurements on D2-D3

Screening for hypoglycemia

BMI

Information

Hormone assays:

C-peptide, IGF-1, leptin, adiponectin

32 weeks 37-38 weeks SssSASA<??>

D28

blood glucose

fasting

BMI: Body mass index, weeks: of gestation, EFW: estimated fetal weight, FHR: fetal heart rate; HC: head circumference.

**Feasibility:**

A systematic survey took place in the delivery room of the Pitié Salpêtrière maternity ward over a 4-month period. It enabled us to identify 58 women giving birth who had had a prepregnancy BMI ≥ 30 kg/m^2^. This represents a mean of 174 women per year for one of the two centers.

The two participating maternity units together have around 4800 deliveries annually. With 8% of the women of child-bearing age having a BMI ≥ 30 kg/m^2^, we can estimate 384 women per year for both centers. This number is consistent with the previous estimate.

**4.3 Steps taken to reduce and avoid bias:**

We will recruit control and obese women simultaneously: when a woman is included in the obese group, a non-obese woman will be included in the control group at the same maternity ward within a maximum of 7 days.

A reference midwife will note among the files of women consulting that day the women who could be included as controls because they meet the inclusion and matching criteria. When she identifies a control subject, she will inform her about the study and the protocol; the physician will collect her consent during the visit. This process will be repeated daily until a control woman is recruited.

Matching will be based initially on the obese woman's age (± 3 years), term of the pregnancy (± 2 weeks) and parity (nulliparous and primi- or multiparous).

The following confounding variables will be taken into account in the data analysis: maternal smoking, maternal diabetes, hypertension during pregnancy, geographic origin, paternal height and BMI, and the infant's sex.

**4.4 Precise description of the study process**

Obese pregnant women will be preselected during their first visit to one of the two maternity units: the women with a prepregnancy BMI ≥ 30 kg/m^2^ who meet no exclusion criteria will be informed about the study protocol. They will receive information forms and will be asked to consent in writing to participate. In accordance with the standard department protocol for this group of at-risk women, a fasting blood glucose test will be ordered. They will be referred for the rest of their prenatal care to a reference obstetrician who will collect their written consent at the next visit, together with the following information:

- geographic origin
- smoking
- height (measured)
- weight at 18 years
- maximum lifetime weight when not pregnant
- minimum weight since the age of 18 years
- prepregnancy weight
- if available, the weight and height at birth and in childhood, from the childhood health records of both parents
- the father's weight, height, and BMI
- familial obesity and diabetes.

The reference physician or midwife will inform and recruit a control woman in the seven days after obtaining written consent, taking into account BMI, age, parity, and gestational age.

Throughout the pregnancy, an obstetric consultation will take place once a month, and the women's weight, blood pressure, and proteinuria will be recorded at each visit.

Women will receive nutritional recommendations at the beginning of the pregnancy: the "Guide for Nutrition during and after Pregnancy", published by the national institute for health prevention and education (INPES) is distributed to all the women and is the reference for nutritional guidelines. Women who were obese before pregnancy or have a history of gestational diabetes will be referred for a consultation with a nutrition expert at the Endocrinology-Diabetology-Nutrition center at Pitié Salpêtrière Hospital.

*Screening for gestational diabetes and blood glucose testing for study participants*

A one-step test will be used to screen for gestational diabetes: an oral glucose tolerance test with 75 g of glucose for 2 hours. The thresholds used will be those proposed by the American Diabetes Association (ADA 2010):

| Oral glucose tolerance test 75 g | Threshold value |
| --- | --- |
| Fasting blood glucose | 0.92 g/L |
| Blood glucose at 1 h | 1.80 g/L |
| Blood glucose at 2 h | 1.53 g/L |

Patients with two values exceeding the threshold will be managed in the diabetes department at Pitié Salpêtrière Hospital for gestational diabetes.

To rule out gestational diabetes during the third trimester of pregnancy and to describe the blood glucose levels of the overall population (BMI<25 or ≥ 30):

- the oral glucose tolerance test will be performed on an outpatient basis between 26 and 28 weeks (except for the women with gestational diabetes at the first screening) and repeated at 32 weeks for the women whose first two screenings are negative.
- Fasting blood glucose will also be tested for all women at the end of pregnancy (between 37 and 38 weeks of gestation) at the same time as the tests required for analgesia at delivery.
- One sample for fructosamine and HbA1c assays (7 mL) will be taken at delivery.

*Management of gestational diabetes*

The women whose screening reveals gestational diabetes will be managed in the diabetes department of Pitié Salpêtrière Hospital. The glycemic goal is to reach fasting and preprandial blood glucose levels ≤ 0.92 g/L (5.1 mmol/L) and a postprandial blood glucose level (2 h after the beginning of the meal) ≤ 1.20 g/L (6.6 mmol/L).

Management will be based on self-monitoring of blood glucose, dietary measures, and the institution of insulin therapy, if necessary, and will take place initially in a day hospital.

- Self-monitoring of blood glucose will take place 6 times daily (before and 2 hours after the beginning of each meal).
- Diet of 1800 calories, with carbohydrate intake balanced throughout the day.
- Medical visit every two weeks with fasting and postprandial tests beforehand and analysis of the patients' blood glucose records.

The effectiveness of the diet will be assessed at each visit or in the day hospital; if ineffective, insulin therapy will be started: either a subcutaneous injection of NPH insulin at bedtime in cases of isolated fasting hyperglycemia; or a combination of a rapid insulin analog and semi-slow insulin at each meal in the case of pre- and postprandial blood glucose levels that exceed the goals.

*The strategy of obstetric monitoring will be adapted according to:*

- the **glycemic control** obtained;
- **fetal weight and physiology** clinical examination, ultrasound to estimate fetal weight (EFW) at 36 weeks of gestation, to be repeated at 38 weeks if there is any suspicion of fetal macrosomia;
- **fetal well-being:** active movements and FHR.

*the target gestational age for delivery is:*

- **for women receiving only dietary management that is successful in meeting glycemic control targets**:
- normal-weight fetus: 40 or 41 weeks of gestation, according to ethnic origin
- macrosomia: 38-39 weeks
  - in cases of insulin treatment:
- 38-39 weeks

In all cases, if fetal weight estimation > 4250 g and abdominal circumference > 370 mm, the delivery will be performed by cesarean.

In the other situations, mode of delivery and gestational age at delivery will be determined by the obstetrician responsible for the patient, depending on the clinical situation. Delivery and immediate neonatal care will be performed according to the department's usual practices.

At arrival in the delivery room, a blood sample will be taken from the mother at the moment the infusion is placed. It will be 7 mL for glycosylated hemoglobin (HbA1c) and fructosamine assays and 7 mL for the leptin and adiponectin assays.

Immediately after delivery and the cutting of the umbilical cord, blood samples will be taken from one of its 2 arteries: a sample into a dry tube (7 mL) for the assays of C-peptide, IGF-1, leptin, adiponectin and its isoforms (appendix 1), a sample for the pH measurement by the automated analyzer in the delivery room, according to the standard technique, and a sample for the blood glucose measurement; the sample will be transported immediately to the biochemistry laboratory of the hospital of birth for measurement.

The usual measurements (weight, height, and head circumference) of the newborns will be collected at birth, as well as the 5- and 10-minute Apgar scores. A complete clinical examination will be performed. Three repeated measurements of tricipital, bicipital, supra-iliac and subscapular skinfolds will be performed with an adipometer, from the first to the third day of life, during the standard clinical examination. Head circumference and height will also be measured twice.

The following data related to neonatal morbidity will be collected: preterm birth (gestational age < 37 weeks), malformations, obstetric injuries (fractures, brachial plexus, etc.), perinatal asphyxia defined by a 5-min Apgar score < 7 and a cord blood pH < 7.2 mmol/L, and respiratory distress, that is, any respiratory difficulty requiring assistance after 30 minutes of life.

Screening for neonatal hypoglycemia is routinely performed for newborn infants of obese or diabetic mothers and in dystrophic newborns (birth weight at or below the 10th percentile or at or above the 90th percentile). Capillary blood glucose will be tested at 1 hour of life and then every 3 hours for at least 24 h or until there are 3 consecutive capillary blood glucose results > 3.3 mmol/L. The onset of hypoglycemia (< 2.5 mmol/L) will require appropriate management according to a pre-established protocol. The capillary blood glucose will be tested in the baby's crib, with a glucometer measuring the drop of blood from a small puncture in the heel. In normal-weight infants (birthweight ranging between the 10th and 90th percentiles) of mothers with a pregestational BMI in the normal range (18.5 to 24.9 kg/m^2^) with negative results from the diabetes screening, capillary blood glucose will not be tested routinely, consistent with standard practice. The episodes of clinically revealed hypoglycemia will be recorded.

Newborns with a neonatal disease requiring hospitalization will be managed, depending on its severity, in the neonatology unit at Pitié-Salpêtrière or in the neonatal intensive care unit or the neonatology department at Trousseau Hospital (Paris 12).

The newborns' weight curve and a specific follow-up of their food in the maternity ward will be recorded.

**4.5 Planned duration of individual participation and description of the trial calendar:**

**Duration of individual study participation** = 9 months (duration of prenatal care, delivery, and the neonatal period (by definition, up to 28 days of life if necessary)

**Planned study duration** = 36 months.

**Duration of inclusion**: 27 months

**Trial calendar:**

| Mother | | | | | |  | Newborn | |
| --- | --- | --- | --- | --- | --- | --- | --- | --- |
|  | Pregnancy  Weight, BP, proteinuria: monthly | | | | | Delivery | Postpartum period | |
|  |  | | | | |  | 3-7 days | |
| Date | 1st visit | 2nd visit | 24-28 weeks | 32 weeks | 37 weeks |  | D0 | D2-3 |
| Procedures | BMI  BP reading  information  orientation | Consent to inclusion  Weight  BP reading | Weight  BP reading | Ultrasound: EFW |  | Weight, height, head circumference  Apgar  Cord blood* | Examination  clinical | Clinical examination  Skinfolds*  Weight, height, head circumference  Feeding |
| Samples |  | Blood glucose  fasting (for the obese women) | Oral glucose tolerance test 75 g. | Oral glucose tolerance test 75 g*. | Fasting blood glucose* | **Mother:**  Fructosamine*  HbA1c*  Leptin*, adiponectin*  **Newborn (cord):**  pH, blood glucose*  C-peptide* IGF-1*  Leptin*, adiponectin* | Capillary blood glucose | |

* : specific examinations for the study

Diabetes screening tests (oral glucose tolerance test 75 g) and fasting blood glucose tests will performed on an outpatient basis. The samples at delivery will be taken in the maternity ward (see appendix 2).

**4.6 Description of rules for temporary or permanent stopping:**

**- of a person from study participation:**

Only the withdrawal of consent can lead to a person's withdrawal from participation in the study:

**- from a part or all of the study:**

There is no justification for stopping the study.

**4.7 Data to be collected directly in the case report forms:**

- Maternal data collected at inclusion by questions about medical history and clinical examination
- Data from pregnancy follow-up:
- Monthly clinical data collection: weight, BP, proteinuria
- Results and dates of diabetes screening: fasting blood glucose, blood glucose values after oral glucose tolerance test 75 g during the second and third trimesters.
- Diabetes management: diet or insulin
- Results of ultrasound and fetal heart rate: fetal growth, malformations, vitality.
- Neonatal data:
- Apgar, cord pH
- Weight, height, head circumference
- Perinatal events: mortality, malformations, obstetric trauma, perinatal asphyxia, respiratory distress, preterm delivery, hypoglycemia
- Blood glucose values for the first 24 hours and beyond
- Hypoglycemia, blood glucose values
- Feeding during the neonatal period
- Laboratory data:
- for the mother: HbA1c, fructosamine, leptin, adiponectin
- for the newborn: cord blood glucose, C-peptide, IGF-1, leptin, adiponectin

**5. Selection and exclusion of study subjects**:

**5.1 Inclusion criteria for subjects agreeing to participate in this study:**

BMI ≥30 kg/m^2^ or between 18.5 and 24.9 kg/m^2^.

Pregnant women consulting at the maternity ward before 18 weeks.

Age ≥ 18 years and ≤ 40 years

Singleton pregnancy

Absence of constitutional or progressive  disease other than obesity: connective tissue diseases, chronic inflammatory diseases, epilepsy, psychiatric disease, tumor…

Provision of written informed consent.

**5.2 Criteria for exclusion of study subjects:**

BMI ≤ 18.5 kg/m^2^ or between 25 and 29.9 kg/m^2^.

Pregnant women coming for their first visit after 18 weeks

Age ≥ 18 years or ≤ 40 years

Multiple pregnancy

Constitutional or progressive disease other than obesity.

Patients who have undergone "bypass" or gastroplasty (ring) surgery for obesity.

Obesity due to genetic disease or secondary to intracranial lesions or pituitary radiation therapy.

Non-stabilized endocrine disease

Diabetes known before the pregnancy

No national health insurance coverage.

**5.3 Procedure for early withdrawal from the study or for exclusion of a study subject, and procedure for their follow-up:**

This study does not require any particular procedure in the case of a participant's withdrawal or exclusion from the research.

**6. Treatment administered to subjects agreeing to participate in this study:**

No treatment is necessary for the performance of this study.

All medication is authorized during this study, except for treatments begun before pregnancy for the treatment of a constitutional or progressive disease and for potentially teratogenic drugs.

**7. Efficacy assessment:**

**Not applicable**

8. Safety assessment:

No adverse effects specific to this study are expected. The specific maternal samples will be taken when other samples are being collected or when an infusion is being placed. The supplementary oral glucose tolerance test during the third trimester presents no special risk. The only study-specific samples for the infant will be taken from the umbilical cord after delivery and after it has been cut.

**9. Adverse effects:**

No adverse effects are expected.

**10. Statistics and data management:**

**10.1 Description of the statistical methods to be used, including the timing of any planned interim analyses.**

The study design plans to include two groups of women, obese (BMI ≥30 kg/m^2^) and non-obese (18.5 ≤BMI< 25 kg/m^2^).

- *Principal statistical analyses*

The principal analysis will estimate the role of maternal obesity on the newborn's body composition. We will seek specially to differentiate the attributable portion associated with obesity from that associated with diabetes. The outcome measures will be the sum of the skinfold measurements, the ponderal index, and the birth weight. We will use a covariance analysis model. Besides obesity and diabetes (no diabetes, diabetes treated by diet only, and diabetes treated by insulin), the following potential confounding factors will be introduced into the model: maternal age, hypertension during pregnancy, smoking, parity, newborn's sex, geographic origin, paternal BMI, gestational age at birth, height of father and mother, and the study center. Fetal deaths will be excluded from the analysis.

- *Secondary analyses*

- The respective roles of obesity and diabetes on the functioning of the newborn's adipose tissue, assessed by the cord blood leptin and adiponectin assays, will be estimated by analysis of variance.

- The role of growth factors such as IGF-1 and insulin (the secretion of which will be estimated by the C-peptide assay) on the newborn's body composition will be estimated by linear regression.

- The relations between the maternal metabolic indicators (leptin, adiponectin, glycosylated hemoglobin, and fructosamine) and the newborn's fat mass (quantity and functioning) on the one hand, and the newborn's growth factors (C-peptide and IGF-1), on the other hand, will also be studied by linear regression.

- The rate of hypoglycemia developing in the 48 hours after the birth of infants of obese mothers will be estimated with its 95% confidence interval.

- The rates of perinatal events in the two groups will be compared with Fisher's exact test.

- *Calendar of analyses*

No interim analysis is planned. The analysis will take place 3 years after the study begins.

**10.2 Planned number of study subjects, and planned number of subjects at each study center, with its statistical justification.**

The primary outcome measures of the study are the ponderal index and sum of skinfolds at birth. For the skinfolds, the study by Sewell et al (Am J Obstet gyneco) compared 76 overweight women (BMI ≥ 25 kg/m^2^) with 144 women with a BMI < 25 kg/m^2^. The skinfold thickness measurements at the tricipital and subscapular levels showed differences, respectively, of 0.4 mm (standard deviation of 0.95 mm) and 0.5 mm (SD 1.2), for an effect size for the two measurements of 0.42. No specific data for obese women have been presented. We have no study of the ponderal index from which we can base any hypothesis to calculate sample size. We would like to be able to show an effect size of at least 0.3 for this criterion.

To obtain a study power of 90% to demonstrate an effect size of 0.3, with an α (type 1) risk of 5%, the analysis should include a total of 470 women (235 in each of the two groups). The rate of loss to follow-up before delivery is estimated at 10%, that of fetal death at 2% (the fetal deaths will be excluded from the analyses of the anthropometric data). Because the groups are matched, we estimate the rate of women who cannot be evaluated for the principal analysis at 24%. It is therefore necessary to include 309 women in each group, for a total of 618 women.

**10.3 Αlpha (type 1) risk**

All tests will be two-sided and will have threshold of significance of 5%.

**10.4 Statistical criteria for halting the study**

No statistical criterion for stopping the study is defined.

**10.5 Method for taking missing, unused, or invalid data into account** .

The subjects for whom outcome measures are not collected will be excluded from the analyses of this criterion.

**10.6 Management of modifications to the analysis plan of the initial strategy.**

All substantial modifications to the initial analysis plan will be justified in the final study report.

**10.7 Selection of subjects to be included in the analyses**

Fetal deaths will be excluded from the analyses of the anthropometric data but will be considered for the analyses of perinatal events.

**10.8 Data management**

The clinical research unit of Pitié-Salpêtrière/Charles Foix Hospitals will be responsible for managing the study data. The data will be collected in electronic case report files. The data file will be reported the CNIL (National Data Protection Authority).

**10.9 Supervisor of data analysis and software**

N Query software was used to plan the study. The analyses will be performed with SAS software, version 8.2 (SAS Institute, Cary, NC), under the supervision of the Pitié-Salpêtrière/Charles Foix clinical research unit.

**11. Budget justification:**

Request for the following posts, to be shared for the two study centers:

- One FTE midwife, whose tasks will be to:

- Identify patients eligible for the study, especially control subjects, during prenatal care visits;
- Inform eligible patients about the research project before the physician requests their consent;
- Ensure that the tests required for the study are ordered (oral glucose tolerance test and fasting blood glucose);
- Spot the records of women included in the study to identify them rapidly on arrival in the delivery room so that the required tests are performed.

- A clinical research technician (4/5 time), whose tasks will be to:

- Ensure the logistic organization of the samples (transportation, storage…)
- Complete the case report forms

Cost of hormone assays:

| Assays | Number of points/patients | | Number of patients | Total points | Number of points/kit | Number of kits | Price/kit, before tax | Total price in euros, before tax |
| --- | --- | --- | --- | --- | --- | --- | --- | --- |
| Leptin | 1 | 1 | 618 | 1236 | 80 | 15,45 | 390 | 6025,5 |
| Adiponectin and HMW | 1 | 1 | 618 | 1236 | 21 | 58,86 | 455 | 26 780 |
| IGF-1 |  | 1 | 618 | 618 | 38 | 16,26 | 475 | 7725 |
| C-peptide |  | 1 | 618 | 618 | 180 | 3,43 | 495 | 1 699,5 |
| Cost of reagents |  |  |  |  |  |  |  | **42 230** |

12. Right of access to data and source documents

Persons with direct access to the data in accordance with the legislative and regulatory provisions in force, specifically, articles 1121-3 and R.5121-13 of the Public Health Code (for example, the investigators, those responsible for quality control, the study monitors, clinical research assistants, auditors, and all individuals whose work involves collaboration with trials) shall take all necessary precautions to ensure the confidentiality of information related to any experimental drugs, trials and participants, especially concerning their identity and the results. The data collected by these persons during quality controls or audits shall be made anonymous.

**13. Quality control and assurance**

**The study shall be overseen according to the sponsor's standard operating procedures.**

The study in the investigational centers and the management of subjects shall comply with the current Declaration of Helsinki and Good Clinical Practices.

The study shall be overseen according to the standard operating procedures of the AP-HP manager. The investigators at each center agree to receive representatives named by the AP-HP for quality control and compliance visits, where appropriate.

14. Legal and ethical considerations

The Sponsor's role is defined by L. 2004-806 dated August 9, 2004. In this study, the AP-HP is the sponsor and its Department of clinical research and development is responsible for fulfilling these regulatory tasks.

**14.1 Request for authorization from AFSSAPS.**

To be allowed to start the study, the AP-HP must, as sponsor, submit an application for authorization from the competent authority, AFSSAPS.

**14.2 Request for an opinion from** the Patient Protection Committee

In accordance with article L.1123-6 of the Public Health Code, the study protocol shall be submitted by the sponsor to a Patient Protection Committee. The sponsor shall notify the competent authority of the committee's opinion before the study begins.

**14.3 Modifications**

The DRCD will be informed of all plans to modify the protocol by the investigator-coordinator.

**14.4 CNIL Declaration**

A report to the CNIL shall be made.

**14.5 Information note and Informed consent**

Each patient shall receive a duplicate of the consent form. The investigator shall verify that the subject understands the implications of participation in the trial and shall receive her written consent (Act N°88-138, Art L.209.9).

The patient information sheets and the informed consent forms provided to the patients are attached hereto, as appendices 2a and 2b.

15. Data treatment and storage of documents and data related to the research

The documents specific to this study shall be stored by all parties for a period of 15 years after the end of the study*.*

This indexed archive shall include:

- Copies of the letter of authorization from AFSSAPS and of the CPP's mandatory opinion
- The successive versions of the protocol (identified by version n° and date),
- Copies of all correspondence with the sponsor,
- The signed consent forms of each subject, with the inclusion list or register
- All completed, validated case report forms for all subjects,
- All appendices specific to the study,
- The final study report
- The database on which the statistical analysis was performed.

**16. Funding and insurance.**

Funding is being requested from the regional Hospital Clinical Research Program for 2009. An evaluation of the costs is included in the appendix.

**17. Rules relative to publication**

The final study report will be written jointly by the principal investigator and the biostatistician. The first authors of any publications shall be people who actually participate in developing the protocol, implementing it, and drafting its results. This report will be submitted to each of the participating center for their opinion.

AP-HP will be the owner of the data from this project and no use by or transmission to a third party may occur without its prior approval.

Assistance Publique-Hôpitaux de Paris will be mentioned as the sponsor of this biomedical research and source of financial support where appropriate. The terms "Assistance Publique-Hôpitaux de Paris" must appears in the authors' address.

**18. List of appendices**

**Appendix 1:** Laboratory tests: procedures for sample-taking, transportation, and storage. Assay techniques

**Appendices 2a and 2b:** Patient information sheet and informed consent document.

**Information sheet for persons participating in the biomedical research study entitled**

**"Impact of maternal obesity on fetal development and perinatal events**

**Analysis of the determinants of fetal growth."**

Sponsor: Assistance Publique Hôpitaux de Paris

Madam,

This document is intended to provide you with information about the study that we are asking you to participate in. Before you agree, it is important that you read this information sheet attentively. The investigator or physician who will speak to you about it is available to answer all of your questions

We inform you that you have the right to agree or refuse to participate in this study.

You can exercise your right to withdraw from the study at any moment.

When you have read this information sheet and received answers to the questions you have by asking the doctor about them, you will be asked, if you agree, to give your written consent by signing the form prepared for this purpose.

- **Aim of the study**

We are asking you to participate in a study aimed at determining the modifications of a newborn's body composition due to the mother's build (obesity); and to analyze the consequences observed in the newborn.

Fetal growth during pregnancy depends on the secretion of some hormones, including, for example, insulin. This secretion varies as a function of the mother's weight and metabolic disorders during pregnancy.

After birth, some hormonal variations can lead to hypoglycemia, which requires monitoring and rapid, individualized management.

- Study plan and procedures

This study, which is being performed prospectively, will study the body composition and hormone levels of newborns according to their mothers' build.

Its aim is to compare two groups of newborns: one group with mothers who are obese, as defined by a prepregnancy body mass index (BMI, weight/height^2^) greater than 30 kg/m^2^, and a control group of mothers with a prepregnancy BMI between 18 and 25 kg/m^2^.

You are being informed of this study at the beginning of your pregnancy, at your first visit to the maternity ward. The obstetrician who will see you throughout your pregnancy will ask for your consent.

This study will take place at the maternity ward. There is no special risk associated with the study, and no direct benefit expected for you or your child. The only differences compared with the standard management are: - an extra screening for gestational diabetes around the 7th month of pregnancy; - a fasting blood glucose test at the end of pregnancy; - blood samples taken at your arrival in the delivery room; - samples of the baby's blood, from the umbilical cord, after it has been cut; - measurement of your baby's skinfolds.

This study will require consideration of some events that might occur during pregnancy, in particular, diabetes. Diabetes screening is performed by an assay (measurement) of blood glucose after ingestion of 75 g of sugar. For this study, it will be performed as usual, during the second trimester of pregnancy (between 5 and 6 months), and one extra time, during the third trimester (around 7 months). If you develop diabetes, you will receive specific care for it from the hospital's diabetes specialists. To verify that your blood glucose level does not change after the screening at 7 months, a fasting blood glucose test will be performed at the same time as you have the blood test necessary for the epidural anesthesia at delivery, as well as a 5-mL blood sample for an insulin assay.

On arrival in the delivery room, at the moment that the infusion needed for delivery is place, a blood sample of 14 mL will be taken to assay some hormones and other components reflecting your metabolism at the end of the pregnancy. These assays will be performed in the weeks after the birth, and the samples will not be stored afterwards.

After the umbilical cord is cut, the person who is performing the delivery will collect 14 ml of blood from the part of the cord attached to the placenta. This procedure will have no consequences for you or the baby. The hormone assays necessary for this study will be performed in the weeks after the birth and will not be stored afterwards.

A sample of the placenta may be collected and stored for 15 years in the Biochemistry, Endocrine, and Oncology laboratory (directed by Pr J Chambaz), located in the Pharmacy Building at Pitié-Salpêtrière Hospital. It will be stored for subsequent studies analyzing the epigenetic consequences to the placenta of maternal build.

To screen for the onset of neonatal hypoglycemia, capillary blood glucose will be measured during the first hours of life in newborns whose mothers are overweight or obese or have diabetes, and in those whose birth weight is very low or very high, according to the standard guidelines. This test will be performed every 3 hours for the first 24 hours of life or until three consecutive samples show normal blood sugar results. It is measured from a drop of blood taken by a small prick of the baby's heel. Hypoglycemia will be treated immediately by appropriate carbohydrate intake.

To assess your baby's body composition, measurements will be made of the thickness of the tricipital, bicipital, subscapular and supra-iliac skinfolds during the standarad pediatric examination between the first and third days of life. It will be in addition to the usual clinical examination and will not cause any particular constraints for your child.

**Study duration**

This study is organized over a 3-year period.

Your participation in the study will cover the duration of your prenatal care, delivery, and of the baby's care immediately after birth, that is, the usual duration of hospitalization in the maternity ward.

- **Sponsor**

The Assistance Publique-Hôpitaux de Paris, which is organizing this biomedical study as its sponsor, has taken out an insurance contract in compliance with legislative requirements, guaranteeing its civil responsibility and that of all participants, from the Gerling France company, located at 111 rue de Longchamp, 75116 Paris, through BIOMEDIC INSURE (tel: 02 97 69 19 19), insurance agent*.*

- **Patient Protection Committee and AFFSAPS**

The Patient Protection Committee of Ile-de-France approved this study, and the competent health authority has authorized its implementation. It is possible that this study will be stopped, if the circumstances so require, by the sponsor or at the request of the public health authorities.

- - **Confidentiality**

In the framework of the biomedical study in which the Sponsor, Assistance publique-Hôpitaux de Paris, has asked you to participate, your personal data and those of your child will be processed so that we can analyze the results of the research project that we have described to you, together with its aims.

For this purpose, the medical data concerning you and the data about your geographic origin will be transmitted to the study sponsor or to people or companies acting for it, in France. These data will be identified by a code number and your initials. These data may also be transmitted, in conditions ensuring their confidentiality, to the French public health authorities and to other entities of Assistance publique–Hopitaux de Paris. In compliance with the provisions of the CNIL (law related to information technology, files, and freedom), you have a right of access and rectification to these data. You also have a right to oppose the transmission of data covered by medical confidentiality that might be used and processed in this study.

You can also have access, directly or through the physician of your choice, to all medical data concerning you, in accordance with article L. 1111-7 of the Public Health Code. These rights can be exercised through the physician who is following you as part of the study and who knows your identity.

- - **Conditions of Participation**

Your participation and that of your child in this biomedical study will not involve any additional expenses for you.

Nonetheless, to be able to participate in this study, you must be covered by a CNAM-TS health insurance.

If you do not agree to the collection of data about your child, or to the taking of samples of cord blood, you cannot participate in this study.

This study does not entail any exclusion period should you wish to participate in another biomedical study after your participation in his research study.

**Informed consent form**

**Consent form to participate in biomedical research**

**for parents or others with parental authority over a child**

I, the underssigned

M^e^, M^lle^, (*Last name, first name*) ……………………………………………

**Agree freely and voluntarily to participate in the biomedical study entitled** "**Impact of maternal obesity on fetal development and perinatal events.** Analysis of the determinants of fetal growth.",

which the Assistance Publique - Hôpitaux de Paris is sponsoring and which was proposed to me by

Doctor (*Last name, first name, telephone*)……………………………………………………………………………..…. …………………………………………………….…, a physician in this study.

On the understanding that:

- The physician who explained this study to me and answered my questions, informed me that I am free to agree or refuse to participate in this study.

- I can communicate with the physician during or after the conclusion of this study about information he or she has about my health,

- I understand from the information sheet that was given to me that to be able to participate in this research, I must be covered by health insurance by CNAM-TS. I confirm that this is the case,

- I can withdraw my consent to participation in this study at any moment, regardless of my reasons and with no liability. If I choose to do so, I will inform the physician. The fact that I choose to stop participating in this study will not affect or harm in any way my relations with this physician,

- If I want, at its conclusion, the physician will inform me of the overall results of this study,

- My consent does not in any way release the physician or the sponsor from any of their responsibilities and I conserve all of our rights guaranteed by law.

*- I have noted that the right of access provided by the CNIL (law of 6 January 1978 related to information technology, files and freedom (art. 39)) can be exercised at any moment by contacting the physician who is following me up in the study and who knows my identity. I can exercise my right of rectification and opposition by contacting this physician, who will contact the study sponsor.*

- *I agree to/refuse* the collection of data concerning my child*
- *I agree to/refuse* the sampling of cord blood after my child's birth.*
- *I agree to/refuse* the conservation of a placenta sample after my child's birth.*
- *(*) cross out the inapplicable words*

***Mother Father***

Signed in , Signed in  ,

Last name, first name Last name, first name

Signature Signature

**Cross out the inapplicable terms*

Signature of the physician who attests to have fully explained to the person signing this form the aim, modalities, and potential risks of this study.

Date: Signature
