# Supplementary material for: Effect of maternal obesity on birthweight and neonatal fat mass: A prospective clinical trial
Source: PLoS One. 2017 Jul 27;12(7):e0181307. doi: 10.1371/journal.pone.0181307 (PMC5531500; doi:10.1371/journal.pone.0181307)
Supplement: S2 File — (DOC) [file pone.0181307.s002.doc]

# PHRC 2009 - APPEL D'OFFRES REGIONAL

# DOSSIER DE DEMANDE

**TITRE DE LA RECHERCHE :**

**Impact de l’obésité maternelle sur le développement fœtal et les évènements périnataux.**

**Analyse des déterminants de la croissance fœtale.**

**Investigateur Principal :**  Pr MITANCHEZ

Service de néonatologie

Hôpital Armand Trousseau

26 avenue du Dr Arnold Netter

7501é PARIS FRANCE

Tél : + 33 1 44 73 61 91 /Fax : + 33 1 44 73 68 92

Email : delphine.mitanchez[@trs.aphp.fr](mailto:jean-philippe.collet@psl.aphp.fr)

**Promoteur :** Assistance Publique - Hôpitaux de Paris

DRCD – Hôpital St Louis

Chef de Projet :

Assistant chef de projet :

**URC :** URC Pitié-Salpêtrière – Charles-Foix

Pr A. Mallet

GH Pitié-Salpêtrière

47, Boulevard de l’hôpital

75013 PARIS France

Tél : + 33 1 42 16 16 53

Email : [urc.pslcfx@psl.aphp.fr](mailto:urc.pslcfx@psl.aphp.fr)

**TABLE DES MATIERES**

[**1. Informations générales :** 4](#__RefHeading___Toc215460090)

[**1.1 : Titre complet de la recherche :** 4](#__RefHeading___Toc215460091)

[**1.2 : Nom et coordonnées du promoteur :** 4](#__RefHeading___Toc215460092)

[**1.3 : Noms et qualités des personnes autorisées à signer le protocole et ses modifications éventuelles au nom du promoteur :** 4](#__RefHeading___Toc215460093)

[**1.4 : Nom, qualité et coordonnées du responsable de la recherche au niveau du promoteur :** 4](#__RefHeading___Toc215460094)

[**1.5 : Noms et qualités des investigateurs :** 4](#__RefHeading___Toc215460095)

[**2. Justification scientifique et description générale de la Recherche :** 5](#__RefHeading___Toc215460096)

[**2.1. Résumé des résultats des essais non cliniques et des essais cliniques disponibles et pertinents au regard de la recherche biomédicale concernée :** 5](#__RefHeading___Toc215460097)

[**2.2. Résumé des bénéfices, le cas échéant, et des risques prévisibles et connus pour les personnes se prêtant à la recherche :** 8](#__RefHeading___Toc215460098)

[**2.3 Description de la population à étudier :** 8](#__RefHeading___Toc215460099)

[**2.4 Références à la littérature scientifique** 8](#__RefHeading___Toc215460100)

[**3. Objectifs de la recherche :** 11](#__RefHeading___Toc215460101)

[**4. Conception de la recherche :** 11](#__RefHeading___Toc215460102)

[**4.1 Critères d’évaluation principaux et des critères d’évaluation secondaires :** 11](#__RefHeading___Toc215460103)

[**4.2 Méthodologie et faisabilité de la recherche:** 12](#__RefHeading___Toc215460104)

[**4.3 Mesures prises pour réduire et éviter les biais :** 13](#__RefHeading___Toc215460105)

[**4.4 Description précise du déroulement de la recherche** 13](#__RefHeading___Toc215460106)

[**4.5 Durée prévue de participation des personnes et description de la chronologie de l’essai:** 15](#__RefHeading___Toc215460107)

[**4.6 Description des règles d’arrêt définitif ou temporaire :** 17](#__RefHeading___Toc215460108)

[**4-7 Données à recueillir directement dans les cahiers d’observation :** 17](#__RefHeading___Toc215460109)

[**5. Sélection et exclusion des personnes de la recherche** : 17](#__RefHeading___Toc215460110)

[**5.1 Critères d’inclusion des personnes qui se prêtent à la recherche :** 17](#__RefHeading___Toc215460111)

[**5.2 Critères de non-inclusion des personnes qui se prêtent à la recherche :** 17](#__RefHeading___Toc215460112)

[**5.3. Procédure d'arrêt prématuré de la recherche ou d'exclusion pour une personne de la recherche et procédure de suivi de la personne :** 18](#__RefHeading___Toc215460113)

[**6. Traitement administré aux personnes qui se prêtent à la recherche :** 18](#__RefHeading___Toc215460114)

[**7. Evaluation de l'efficacité :** 18](#__RefHeading___Toc215460115)

[**8. Evaluation de la sécurité :** 18](#__RefHeading___Toc215460116)

[**9. Effets indésirables :** 18](#__RefHeading___Toc215460117)

[**10. Statistiques et gestion des données :** 18](#__RefHeading___Toc215460118)

[**10.1. Description des méthodes statistiques prévues, y compris du calendrier des analyses intermédiaires prévues.** 18](#__RefHeading___Toc215460119)

[**10.2. Nombre prévu de personnes à inclure dans la recherche, et nombre prévu de personnes dans chaque lieu de recherches avec sa justification statistique.** 19](#__RefHeading___Toc215460123)

[**10.3. Risque de première espèce** 19](#__RefHeading___Toc215460124)

[**10.4. Critères statistiques d'arrêt de la recherche** 20](#__RefHeading___Toc215460125)

[**10.5. Méthode de prise en compte des données manquantes, inutilisées ou non valides**. 20](#__RefHeading___Toc215460126)

[**10.6. Gestion des modifications apportées au plan d'analyse de la stratégie initiale.** 20](#__RefHeading___Toc215460127)

[**10.7. Choix des personnes à inclure dans les analyses** 20](#__RefHeading___Toc215460128)

[**10.8. Gestion des données** 20](#__RefHeading___Toc215460129)

[**10.9. Responsable de l’analyse des données et logiciels de travail** 20](#__RefHeading___Toc215460130)

[**11. Justification du budget :** 20](#__RefHeading___Toc215460131)

[**12. Droit d'accès aux données et documents source** 21](#__RefHeading___Toc215460132)

[**13. Contrôle et assurance de la qualité** 21](#__RefHeading___Toc215460133)

[**14. Considérations légales et éthiques** 21](#__RefHeading___Toc215460134)

[**14.1 Demande d’autorisation auprès de l’AFSSAPS.** 21](#__RefHeading___Toc215460135)

[**14.2 Demande d’avis au Comité de Protection des Personnes** 21](#__RefHeading___Toc215460136)

[**14.3 Modifications** 21](#__RefHeading___Toc215460137)

[**14.4 Déclaration CNIL** 21](#__RefHeading___Toc215460138)

[**14.5 Note d’information et Consentement éclairé** 21](#__RefHeading___Toc215460139)

[**15. Traitement des données et conservation des documents et des données relatives à la recherche** 22](#__RefHeading___Toc215460140)

[**16. Financement et assurance.** 22](#__RefHeading___Toc215460141)

[**17. Règles relatives à la publication** 22](#__RefHeading___Toc215460142)

[**18. Liste des annexes** 22](#__RefHeading___Toc215460143)

**PROTOCOLE DE LA RECHERCHE**

**1. Informations générales :**

**1.1 : Titre complet de la recherche :**

**Impact de l’obésité maternelle sur le développement fœtal et les évènements périnataux.**

**Analyse des déterminants de la croissance fœtale.**

**- Titre abrégé : Nouveau-nés de mère obèse.**

**- Numéro de code du protocole attribué par le promoteur :**

**- Numéro de version :**

**- Date du protocole :** 26/11/2008

**1.2 : Nom et coordonnées du promoteur :**

Assistance Publique - Hôpitaux de Paris

DRCD – Hôpital St Louis

**- Représentant légal dans la Communauté européenne :**

**- Organisme prestataire chargé du suivi de l’essai :**

**1.3 : Noms et qualités des personnes autorisées à signer le protocole et ses modifications éventuelles au nom du promoteur :**

**1.4 : Nom, qualité et coordonnées du responsable de la recherche au niveau du promoteur :**

**1.5 : Noms et qualités des investigateurs :**

Responsable : Pr MITANCHEZ Delphine, PU-PH, chef de service, Néonatologie, hôpital Armand Trousseau et Pitié Salpêtrière, GHU-Est.

Nom et qualité des autres personnes qui collaborent à cette recherche : M Dommergues (PU-PH),

J Nizard (PHU), C Renaud (PH), I Demontgolfier (PH), A Wallet (PH), M Moreno (PHC), JM Jouannic (PU-PH), C De Carne (CCA), A Basdevant (PU-PH), C Ciangura (CCA), S Jaqueminet (PH), A Grimaldi (PU-PH), C Sachon, (PH), JM Lacorte (MCU-PH), C Coussieu (MCU-PH), V Frochot (AHU), MA Charles (ATT, DR).

**- Lieu de recherche :**

Maternité, Hôpital Pitié-Salpêtrière. 47, boulevard de l’hôpital. 75 013 PARIS.

Pôle de Périnatalité, Hôpital Armand Trousseau 26 avenue du Dr Arnold Netter, 75012 PARIS

**2. Justification scientifique et description générale de la Recherche :**

**2.1 Résumé des résultats des essais non cliniques et des essais cliniques disponibles et pertinents au regard de la recherche biomédicale concernée :**

Au cours des dernières décennies, une proportion grandissante de femmes débutant leur grossesse est en état de surcharge pondérale. En France, l’enquête épidémiologique nationale sur l’obésité et le surpoids publiée en septembre 2006 (enquête ObEpi) a confirmé l’augmentation de l’incidence de l’obésité déjà constatée au cours des années antérieures [1]. Le surpoids concerne près de 14 millions de français. On note une hausse plus rapide et plus importante de la prévalence chez les femmes comparé aux hommes (+64% versus +40%) : 23% des femmes sont en surpoids et 13% sont obèses. Ces femmes sont exposées à une morbidité importante et pour celles en âge de procréer à des complications au cours de la grossesse.

Les deux complications de l’obésité les plus fréquentes durant la grossesse sont l’hypertension artérielle avec le risque de pré-éclampsie et le diabète gestationnel. L’obésité maternelle est aussi associée à une fréquence accrue de césariennes, de décès fœtal ou néonatal, de malformations, de naissance prématurée et à des complications plus fréquentes chez le nouveau-né, liée en partie à la macrosomie [2-6].

Les particularités néonatales des nouveau-nés de mère obèse sont mal connues car les données de la littérature relèvent pour la plupart d’études obstétricales qui ne renseignent que succinctement sur la morbidité néonatale immédiate à partir des données relevées en salle de naissance. Il n’existe pas d’étude sur ce sujet menée par des équipes de néonatologistes s’attachant aux premiers jours de vie. Les complications fœtales et néonatales décrites dans le cadre des grossesses de femmes obèses semblent de prime abord analogues à celles décrites en cas de diabète au cours de la grossesse. Les données actuelles ne permettent pas de déterminer la pathologie spécifiquement reliée à l’obésité maternelle indépendamment du diabète. Pourtant, il semble que les modifications du milieu intra-utérin secondaires à l’obésité maternelle puissent à elles seules entraîner des conséquences fœtales et néonatales spécifiques. C’est ce qui est illustré par les données sur les malformations. Chez les femmes enceintes obèses, le risque de malformations fœtales semblent être augmenté, et concerne essentiellement les anomalies du tube neural mais pourrait également atteindre d’autres organes comme le cœur, le tube digestif, le poumon [7-11]. Certains attribuent ces malformations à l’association fréquente de l’obésité et du diabète, mais d’autres études ont montré que l’obésité est un facteur de risque indépendant de malformations fœtales [12]. Ainsi, on retrouve une association avec le risque de hernie de coupole diaphragmatique et d’omphalocoele, qui n’est pas classiquement rapportée dans le diabète [8].

Certaines études ont montré que les nouveau-nés de mère obèse ont une augmentation du poids de naissance en rapport avec l’IMC (indice de masse corporelle) maternel pré-conceptionnel et la prise de poids pendant la grossesse indépendamment du diabète [13, 14]. L’augmentation du poids de naissance des nouveau-nés de femmes obèses sans diabète serait liée uniquement à une augmentation de la masse grasse mais non de la masse maigre [15]. La composition corporelle des nouveau-nés peut être évaluée à partir de mesures anthropométriques qui permettent une estimation fiable de la masse maigre et de la masse grasse [16]. Celles-ci comportent le poids et la taille à partir desquelles sont calculés l’IMC (poids/taille^2^) et l’index pondéral (poids/taille^3^), le périmètre crânien et l’épaisseur des plis cutanés tricipital, bicipital, supra-iliaque et sous-scapulaire. Il existe une bonne corrélation chez le nouveau-né entre l’estimation de la masse grasse par la mesure de ces plis cutanés et la mesure par absorptiométrie [17].

Les mécanismes à l’origine de l’excès de croissance fœtale au cours des grossesses de femmes obèses ne sont pas connus. On peut supposer que des modifications proviennent d’altérations dans le métabolisme maternel. Ainsi, plusieurs auteurs ont montré l’influence de la qualité des apports nutritionnels maternels, en particuliers pour les macronutriments (protides, glucides et lipides), sur la croissance fœtale [18, 19]. Une étude récente évoque le lien entre les apports nutritionnels maternels au cours de la grossesse et la modification de la production d’hormones du métabolisme, comme la leptine, l’adiponectine et la résistine. Or, certaines de ces hormones stimulent le transport placentaire de nutriments [20]. Ainsi, il existerait un lien entre le surpoids et l’augmentation d’hormones du métabolisme chez la mère ce qui induirait un excès de croissance fœtale par augmentation du transport placentaire des nutriments [21].

On connait actuellement plusieurs facteurs fœtaux qui interviennent sur la croissance fœtale, mais on ne sait pas quelle est l’influence de l’obésité maternelle sur l’expression de ces facteurs.

L’insuline est un facteur de croissance essentiel pour le fœtus. La croissance excessive des nouveau-nés de mère diabétique est ainsi attribuée à l’hyperinsulinisme fœtal secondaire à l’hyperglycémie maternelle. L’effet trophique de l’insuline résulte de plusieurs mécanismes : l’insuline stimule l’entrée et l’utilisation des nutriments par les tissus insulino-sensibles (dont le tissu adipeux), elle a un effet mitogène direct et enfin, elle interagit avec le système des IGF (Insulin-Like Growth Factor) en stimulant la production d’IG-1 [22]. Les IGF jouent un rôle déterminant dans la régulation de la croissance fœtale et post-natale. IGF-2 est impliqué principalement dans la croissance fœtale, alors que son homologue, IGF-1, est impliqué durant la période fœtale et post-natale. Ces deux facteurs de croissance agissent sur le même récepteur tyrosine kinase, IGF-R, et sont les médiateurs essentiels de la GH. Le taux d’IGF-1 est diminué en cas de retard de croissance et augmenté chez les nouveau-nés macrosomes de mère diabétique. IGF-1 partage une homologie de structure avec la proinsuline et possède des effets métaboliques analogues à l’insuline [23].

La leptine est une protéine essentiellement produite par le tissu adipeux mais aussi par le placenta. Le tissu adipeux fœtal est une source importante de leptine dont la production suit le développement de ce tissu : le taux de leptine est bas au cours de la première moitié de la grossesse puis augmente fortement au cours du troisième trimestre. Sa production pourrait être régulée par l’insuline, mais également par l’hypoxie fœtale [24]. La leptine maternelle ne traverse pas le placenta. La leptine placentaire est relarguée en grande majorité chez la mère, seule une très faible fraction passe chez le fœtus [25]. Certains auteurs considèrent que le taux de leptine à la naissance n’est que le reflet de la masse adipeuse [26]. D’autres en revanche, considèrent que c’est un facteur important pour la croissance et le développement du fœtus, son action étant indépendante de l’insuline et d’IGF-1 [27-29]. Son mécanisme d’action sur la croissance fœtal demeure toutefois inconnu.

L’adiponectine est une autre protéine secrétée exclusivement par le tissu adipeux. Elle est paradoxalement diminuée chez le sujet obèse et augmente au cours de l’amaigrissement. L’adiponectine est un modulateur important de l’action de l’insuline et du métabolisme du glucose, deux éléments régulateurs de la croissance fœtale. Cela fait de l’adiponectine un facteur potentiel de la régulation de la croissance fœtale. Différentes études ont analysé le profil d’expression de l’adiponectine chez le nouveau-né en fonction éventuellement de la croissance in utero. Les résultats sont souvent contradictoires, mais il ressort que le profil d’expression est différent de celui observé chez l’adulte. En effet, les taux chez le nouveau-né sont élevés par rapport à ceux de l’adulte [30, 31]. Il existerait une corrélation positive entre le poids de naissance et le taux d’adiponectine dosé au sang du cordon chez le prématuré, le nouveau-né avec retard de croissance et le nouveau-né eutrophe à terme [32]. En revanche, ce taux serait diminué chez le nouveau-né macrosome [33]. Pour certains, contrairement à l’adulte, il n’y a pas de relation chez le fœtus entre le taux d’adiponectine et la masse adipeuse [34]. Enfin, une étude récente a montré que l’adiponectine au cordon ne serait corrélée qu’à la taille de naissance [35]. Elle agirait par une augmentation de la sensibilité fœtale à l’insuline et de ce fait une potentialisation de l’effet d’IGF-1. L’adiponectine n’a jamais été étudiée spécifiquement chez le nouveau-né de mère obèse. Par ailleurs, plusieurs isoformes d’adiponectine existent (forme globulaire, forme hexamérique, forme de haut poids moléculaire) [36] dont les variations n’auraient pas la même signification métabolique [37].

Ces différents facteurs de croissance jouent aussi un rôle dans l’homéostasie glucidique. L’hypoglycémie des nouveau-nés de mère diabétique a été beaucoup étudiée et est attribuée à l’hyperinsulinisme qui persiste après la naissance. Cependant, on n’observe pas toujours de relation entre le risque d’hypoglycémie et l’importance de la macrosomie [38]. Une seule étude rapporte une augmentation du risque d’hypoglycémie chez les nouveau-nés de mère obèse [39], et une corrélation positive entre l’IMC maternel au début de grossesse et le taux d’insuline et de peptide-C mesurés au cordon a par ailleurs été retrouvée [38].

Nous proposons une étude chez les nouveau-nés de femme obèse c'est-à-dire avec un IMC ≥ 30, qui isole une population de femmes de poids extrême peu accessibles dans les études en population générale. Nous partons de l’hypothèse que les nouveau-nés de mère obèse ont en moyenne un poids de naissance plus élevé. Nous voulons évaluer les conséquences de l’obésité maternelle :

- sur le poids et le tissu adipeux du nouveau-né,
- la fonctionnalité du tissu du tissu adipeux (production de leptine et d’adiponectine),
- sur les facteurs de croissance fœtale (insuline, IGF-1)
- sur le métabolisme glucidique du nouveau-né.

Nous déterminerons la part de l’obésité et du diabète sur chacun de ces éléments.

**2.2 Résumé des bénéfices, le cas échéant, et des risques prévisibles et connus pour les personnes se prêtant à la recherche :**

Nous proposons une étude qui ne comporte pas de risque pour la mère et l’enfant et dont l’objectif principal n’apporte pas de bénéfice immédiat.

La prise en charge obstétricale de la grossesse sera habituelle. Seul un test supplémentaire pour le dépistage du diabète par HGPO au 3^ème^ trimestre sera réalisé, ainsi que 2 prélèvements sanguins réalisés au moment d’autres prélèvements ou de la mise en place d’une perfusion. Leur volume est faible (2 ml pour le 1^er^ prélèvement, 12 ml pour le 2^ème^).

La prise en charge du nouveau-né n’est en rien modifiée par l’étude. Les prélèvements sanguins spécifiques à l’étude pour les nouveau-nés seront réalisés au sang du cordon.

Les données seront utilisées de façon anonyme.

**2.3Description de la population à étudier :**

La population à étudier concerne les nouveau-nés singletons de mère obèse (indice de masse corporelle (IMC) ≥ 30 kg/m^2^ avant la grossesse) suivie et ayant accouché dans les deux maternités de l’étude, la maternité de l’hôpital Pitié Salpêtrière et la maternité de l’hôpital Trousseau à Paris. La population témoin est constituée de nouveau-nés singletons de mère non obèse (18,5 ≤IMC< 25 kg/m^2^ avant la grossesse) suivie et ayant accouché dans l’un des deux maternités de l’étude.

**2.4 Références à la littérature scientifique:**

1. Charles MA, Eschwege E, Basdevant A. Monitoring the obesity epidemic in France: the obepi surveys 1997-2006. Obesity (Silver Spring) 2008;16(9):2182-6.

2. Callaway LK, Prins JB, Chang AM, McIntyre HD. The prevalence and impact of overweight and obesity in an Australian obstetric population. Med J Aust 2006;184(2):56-9.

3. Cedergren MI. Maternal morbid obesity and the risk of adverse pregnancy outcome. Obstet Gynecol 2004;103(2):219-24.

4. Weiss JL, Malone FD, Emig D, Ball RH, Nyberg DA, Comstock CH, et al. Obesity, obstetric complications and cesarean delivery rate--a population-based screening study. Am J Obstet Gynecol 2004;190(4):1091-7.

5. Kristensen J, Vestergaard M, Wisborg K, Kesmodel U, Secher NJ. Pre-pregnancy weight and the risk of stillbirth and neonatal death. Bjog 2005;112(4):403-8.

6. Cnattingius S, Bergstrom R, Lipworth L, Kramer MS. Prepregnancy weight and the risk of adverse pregnancy outcomes. N Engl J Med 1998;338(3):147-52.

7. Ray JG, Wyatt PR, Vermeulen MJ, Meier C, Cole DE. Greater maternal weight and the ongoing risk of neural tube defects after folic acid flour fortification. Obstet Gynecol 2005;105(2):261-5.

8. Waller DK, Shaw GM, Rasmussen SA, Hobbs CA, Canfield MA, Siega-Riz AM, et al. Prepregnancy obesity as a risk factor for structural birth defects. Arch Pediatr Adolesc Med 2007;161(8):745-50.

9. Watkins ML, Rasmussen SA, Honein MA, Botto LD, Moore CA. Maternal obesity and risk for birth defects. Pediatrics 2003;111(5 Part 2):1152-8.

10. Cedergren M, Kallen B. Maternal obesity and the risk for orofacial clefts in the offspring. Cleft Palate Craniofac J 2005;42(4):367-71.

11. Cedergren MI, Kallen BA. Maternal obesity and infant heart defects. Obes Res 2003;11(9):1065-71.

12. Hendricks KA, Nuno OM, Suarez L, Larsen R. Effects of hyperinsulinemia and obesity on risk of neural tube defects among Mexican Americans. Epidemiology 2001;12(6):630-5.

13. Ehrenberg HM, Mercer BM, Catalano PM. The influence of obesity and diabetes on the prevalence of macrosomia. Am J Obstet Gynecol 2004;191(3):964-8.

14. Okun N, Verma A, Mitchell BF, Flowerdew G. Relative importance of maternal constitutional factors and glucose intolerance of pregnancy in the development of newborn macrosomia. J Matern Fetal Med 1997;6(5):285-90.

15. Sewell MF, Huston-Presley L, Super DM, Catalano P. Increased neonatal fat mass, not lean body mass, is associated with maternal obesity. Am J Obstet Gynecol 2006;195(4):1100-3.

16. Koo WW, Walters JC, Hockman EM. Body composition in neonates: relationship between measured and derived anthropometry with dual-energy X-ray absorptiometry measurements. Pediatr Res 2004;56(5):694-700.

17. Schmelzle HR, Fusch C. Body fat in neonates and young infants: validation of skinfold thickness versus dual-energy X-ray absorptiometry. Am J Clin Nutr 2002;76(5):1096-100.

18. Godfrey K, Robinson S, Barker DJ, Osmond C, Cox V. Maternal nutrition in early and late pregnancy in relation to placental and fetal growth. Bmj 1996;312(7028):410-4.

19. Moore VM, Davies MJ, Willson KJ, Worsley A, Robinson JS. Dietary composition of pregnant women is related to size of the baby at birth. J Nutr 2004;134(7):1820-6.

20. Jones HN, Powell TL, Jansson T. Regulation of placental nutrient transport--a review. Placenta 2007;28(8-9):763-74.

21. Jansson N, Nilsfelt A, Gellerstedt M, Wennergren M, Rossander-Hulthen L, Powell TL, et al. Maternal hormones linking maternal body mass index and dietary intake to birth weight. Am J Clin Nutr 2008;87(6):1743-9.

22. Hill DJ, Petrik J, Arany E. Growth factors and the regulation of fetal growth. Diabetes Care 1998;21 Suppl 2:B60-9.

23. Holt RI, Simpson HL, Sonksen PH. The role of the growth hormone-insulin-like growth factor axis in glucose homeostasis. Diabet Med 2003;20(1):3-15.

24. Hytinantti TK, Koistinen HA, Teramo K, Karonen SL, Koivisto VA, Andersson S. Increased fetal leptin in type I diabetes mellitus pregnancies complicated by chronic hypoxia. Diabetologia 2000;43(6):709-13.

25. Alexe DM, Syridou G, Petridou ET. Determinants of early life leptin levels and later life degenerative outcomes. Clin Med Res 2006;4(4):326-35.

26. Hauguel-de Mouzon S, Lepercq J, Catalano P. The known and unknown of leptin in pregnancy. Am J Obstet Gynecol 2006;194(6):1537-45.

27. Hassink SG, de Lancey E, Sheslow DV, Smith-Kirwin SM, O'Connor DM, Considine RV, et al. Placental leptin: an important new growth factor in intrauterine and neonatal development? Pediatrics 1997;100(1):E1.

28. Wiznitzer A, Furman B, Zuili I, Shany S, Reece EA, Mazor M. Cord leptin level and fetal macrosomia. Obstet Gynecol 2000;96(5 Pt 1):707-13.

29. Vatten LJ, Nilsen ST, Odegard RA, Romundstad PR, Austgulen R. Insulin-like growth factor I and leptin in umbilical cord plasma and infant birth size at term. Pediatrics 2002;109(6):1131-5.

30. Kamoda T, Saitoh H, Saito M, Sugiura M, Matsui A. Serum adiponectin concentrations in newborn infants in early postnatal life. Pediatr Res 2004;56(5):690-3.

31. Pardo IM, Geloneze B, Tambascia MA, Barros-Filho AA. Hyperadiponectinemia in newborns: relationship with leptin levels and birth weight. Obes Res 2004;12(3):521-4.

32. Tsai PJ, Yu CH, Hsu SP, Lee YH, Chiou CH, Hsu YW, et al. Cord plasma concentrations of adiponectin and leptin in healthy term neonates: positive correlation with birthweight and neonatal adiposity. Clin Endocrinol (Oxf) 2004;61(1):88-93.

33. Mazaki-Tovi S, Kanety H, Pariente C, Hemi R, Schiff E, Sivan E. Cord blood adiponectin in large-for-gestational age newborns. Am J Obstet Gynecol 2005;193(3 Pt 2):1238-42.

34. Lindsay RS, Walker JD, Havel PJ, Hamilton BA, Calder AA, Johnstone FD. Adiponectin is present in cord blood but is unrelated to birth weight. Diabetes Care 2003;26(8):2244-9.

35. Inami I, Okada T, Fujita H, Makimoto M, Hosono S, Minato M, et al. Impact of serum adiponectin concentration on birth size and early postnatal growth. Pediatr Res 2007;61(5 Pt 1):604-6.

36. Kadowaki T, Yamauchi T, Kubota N, Hara K, Ueki K, Tobe K. Adiponectin and adiponectin receptors in insulin resistance, diabetes, and the metabolic syndrome. J Clin Invest 2006;116(7):1784-92.

37. Basu R, Pajvani UB, Rizza RA, Scherer PE. Selective downregulation of the high molecular weight form of adiponectin in hyperinsulinemia and in type 2 diabetes: differential regulation from nondiabetic subjects. Diabetes 2007;56(8):2174-7.

38. Soltani KH, Bruce C, Fraser RB. Observational study of maternal anthropometry and fetal insulin. Arch Dis Child Fetal Neonatal Ed 1999;81(2):F122-4.

39. Doherty DA, Magann EF, Francis J, Morrison JC, Newnham JP. Pre-pregnancy body mass index and pregnancy outcomes. Int J Gynaecol Obstet 2006;95(3):242-7.

**3. Objectifs de la recherche :**

**L’objectif principal** de ce projet est de rechercher si les nouveau-nés de mère obèse présentent des modifications de la composition corporelle évaluée sur le poids et la masse grasse à la naissance. On s’attachera particulièrement à différencier le rôle de l’obésité maternelle de celui du diabète.

**Les objectifs secondaires** sont d’étudier :

- les différences de fonctionnalité du tissu adipeux du nouveau-né de mère obèse évaluées par le dosage de la leptine et de l’adiponectine ;
- les relations entre différents facteurs de croissance tels que l’IGF-1 et l’insuline et le poids et la masse grasse du nouveau-né (parce que l'hémolyse est 
  fréquente lors des prélèvements de sang de cordon et que l'insuline ne 
  peu pas être dosée dans ces conditions, l'insuline sera évaluée par 
  le dosage du peptide C qui est en quantité équimolaire).
- les relations entre les paramètres métaboliques maternels (leptine, adiponectine, hémoglobine glyquée HbA1c, fructosamine et glycémie), la masse grasse du nouveau-né (quantité et de fonctionnalité) et les facteurs de croissance du nouveau-né (peptide C et IGF-1) ;
- Les relations entre l’évolution des glycémies du nouveau-né en période postnatale immédiate et les taux d’hormones mesurées au cordon (peptide C, IGF-1, leptine, adiponectine).
- Estimation du taux d’évènements périnataux (mortalité, morbidité) et du taux d’hypoglycémie.

**4. Conception de la recherche :**

**4.1 Critères d’évaluation principaux et des critères d’évaluation secondaires :**

Les **critères d’évaluation principaux** sont :

- Les données anthropométriques des nouveau-nés de mère obèse, c'est-à-dire l’index pondéral, la somme de l’épaisseur des plis cutanés et le poids. Ces paramètres anthropométriques permettent une estimation fiable de l’adiposité du nouveau-né.

Les **critères d’évaluation secondaires** sont :

- Les taux d’hormones impliquées dans la régulation de la croissance fœtale ou reflétant la fonctionnalité du tissu adipeux fœtal, prélevées au sang du cordon: peptide C, l’IGF-1, la leptine, l’adiponectine et ses différentes isoformes.

- Les paramètres métaboliques maternels:

- L’existence ou non d’un diabète et les modalités de traitement
- Le dosage de la fructosamine et de l’HbA1c à l’accouchement (reflétant l’équilibre glycémique maternel moyen au cours des semaines précédant le dosage)
- Les taux de leptine et d’adiponectine maternels à l’accouchement pour caractériser le tissu adipeux maternel.

- L’équilibre glycémique du nouveau-né au cours des premiers jours de vie évalué sur la surveillance des glycémies capillaires.

- Les évènements périnataux : mortalité, malformations, traumatisme obstétrical, asphyxie périnatale, détresse respiratoire, prématurité, hypoglycémie.

**4.2 Méthodologie et faisabilité de la recherche:**

Il s’agit d’une étude observationnelle prospective, exposés-non exposés, bi-centrique.

**Représentation schématique de la recherche**

Naissance

=

J0

Période néonatale

Grossesse

1^ère^ consultation

2^ème^ consultation

consentement

glycémie à jeun

HGPO 75 g

24-28 SA

1 consultation/mois

Prise de poids,TA, protéinurie

HGPO 75g

Echographie

RCF

± prise en charge diabétologie

Prélèvement maternel : HbA1c

fructosamine, leptine, adiponectine

Prélèvement de

sang au cordon

Apgar, pH

Poids taille PC

Mesure plis cutanés à J2-J3

Dépistage des hypoglycémies

IMC

Information

Dosages hormonaux:

peptide C, IGF1, leptine, adiponectine

32 SA 37-38 SA SssSASA

J28

glycémie

à jeun

IMC : indice de masse corporelle, SA : semaines d’aménorrhée, EPF : estimation du poids fœtal, RCF : rythme cardiaque fœtal ; PC : périmètre crânien.

**Faisabilité :**

Un relevé systématique en salle de naissance de la maternité Pitié Salpêtrière a été réalisé durant une période de 4 mois. Il a permis d’identifier 58 femmes venues accoucher avec un IMC ≥ 30 kg/m^2^ en début de grossesse. Cela représente une moyenne de 174 femmes par an pour un des deux centres.

Les deux maternités impliquées dans l’étude réalisent 4800 accouchements par an au total. Si on considère que 8% des femmes en âge de procréer ont un IMC ≥ 30 kg/m^2^, cela permet une estimation de 384 femmes par an sur les deux centres. Ce nombre est en accord avec l’estimation précédente.

**4.3 Mesures prises pour réduire et éviter les biais :**

Le recrutement des femmes témoins est réalisé simultanément à celui des femmes obèses : lorsqu’une femme est inclue dans le groupe des femmes obèses, une femme non obèse est inclue dans le groupe témoin dans la même maternité et dans un délai de 7 jours maximum.

Une sage-femme référente relèvera parmi les dossiers de consultation de la journée les femmes susceptibles d’être incluses comme témoin car répondant aux critères d’inclusion et d’appariement. Lorsqu’elle aura identifié un sujet témoin, elle transmettra l’information sur le protocole et le consentement sera recueilli par le médecin lors de la consultation. Ce processus sera répété chaque jour jusqu’au recrutement du témoin.

L’appariement des sujets témoins est effectué initialement sur l’âge des patientes (± 3 ans), le terme de la grossesse (± 2 SA) et la parité (nullipare et multipare).

Les variables confondantes prises en compte dans l’analyse des données sont : le tabagisme maternel, le diabète maternel, l’HTA pendant la grossesse, l’origine géographique, la taille et l’IMC paternel, le sexe du nouveau-né.

**4.4 Description précise du déroulement de la recherche**

La présélection des femmes enceintes obèses s’effectue lors de la 1^ère^ visite dans l’une des deux maternités: les femmes ayant un IMC pré-gestationnel ≥ à 30 kg/m^2^ sont informées en l’absence de critères d’exclusion, du protocole de recherche. Le formulaire d’information et de recueil du consentement leur sont remis. Selon le protocole habituel du service pour ce groupe de femmes à risque, un dosage de glycémie à jeun sera prescrit. Elles sont orientées pour la suite de leur grossesse vers un obstétricien référent qui recueillera le consentement à la visite suivante, ainsi que les informations suivantes :

- origine géographique
- consommation de tabac
- la taille (mesurée)
- le poids à 18 ans
- le poids maximum au cours de la vie en dehors des grossesses
- le poids minimum depuis l’âge de 18 ans
- le poids avant la grossesse
- si disponible les poids et tailles à la naissance et dans l’enfance à partir du carnet de santé pour le père et la mère
- le poids, la taille et l’IMC du père
- la notion d’obésité ou de diabète familial.

Le consultant/ et ou la sage-femme référent informe et recrute une femme témoin dans les sept jours suivants le recueil du consentement, en tenant compte de l’IMC, l’âge, la parité et le terme de la patiente.

Pendant toute la durée de la grossesse, une consultation obstétricale est effectuée une fois par mois par le consultant référent en précisant le poids mensuel, la PA et la protéinurie des patientes.

Des recommandations nutritionnelles sont transmises en début de grossesse : le « guide nutrition pendant et après la grossesse » mis en place par l’Institut National de Prévention et d’Education pour la Santé est remis à toutes les femmes et sert de référence pour les recommandations nutritionnelles. En cas d’obésité préexistant à la grossesse ou d’antécédent de diabète gestationnel, une consultation en diététique est prévue dans le Pôle d’Endocrinologie-Diabétologie-Nutrition de la Pitié Salpêtrière.

*Dépistage du diabète gestationnel et étude de glycémique des femmes de l’étude*

Pour le dépistage du diabète gestationnel, le test utilisé sera en un temps par une hyperglycémie provoquée par voie orale (HGPO) avec 75gr de glucose sur 2 heures. Les seuils utilisés seront ceux proposés par l’American Diabetes Association (ADA 2010) :

| HGPO 75gr | Valeurs seuil |
| --- | --- |
| Glycémie à jeun | O.92 g/l |
| Glycémie à 1h | 1.80 g/l |
| Glycémie à 2 h | 1.53 g/l |

Les patientes ayant deux valeurs au dessus des valeurs seuil seront prises en charge dans le service de Diabétologie de l’hôpital de la Pitié Salpêtrière pour diabète gestationnel.

Afin d’éliminer le diabète gestationnel lors du troisième trimestre de la grossesse, et de décrire l’ensemble de la population (BMI<25 ou ≥ 30) sur le plan glycémique :

- L’HGPO sera réalisée en ambulatoire entre 26 et 28 SA (sauf pour les femmes présentant un diabète gestationnel au premier dépistage), et répétée à 32 SA pour les femmes dont les deux premiers dépistages seront négatifs ;
- Une glycémie à jeun sera également réalisée pour toutes les femmes en fin de grossesse (entre 37 et 38 semaine d’aménorrhée) au moment de la réalisation du bilan nécessaire à l’analgésie de l’accouchement ;
- Un dosage de fructosamine et d’HbA1c sera prélevé à l’accouchement (volume de prélèvement 7 ml).

*Prise en charge du diabète gestationnel*

Les femmes dépistées pour le diabète gestationnel seront prises en charge dans le service de diabétologie de l’hôpital de la Pitié Salpêtrière. L’objectif glycémique est d’obtenir une glycémie à jeun et préprandiale inférieure ou égale à 0.92g/L (5,1mmol/L) et une glycémie postprandiale (2 h après le début du repas) inférieure ou égale à 1.20 g/L (6,6 mmol/L).

La prise en charge reposera sur l’autosurveillance glycémique, les mesures diététiques et l’instauration d’une insulinothérapie si nécessaire, et se fera initialement en hôpital de jour.

- Réalisation de l’autosurveillance glycémique, 6 fois par jour (avant et 2 heures après le début de chaque repas).
- Régime 1800 calories fractionné en glucides.
- Consultation médicale tous les 15 jours avec réalisation au préalable d’une glycémie à jeun et post prandiale et analyse du carnet glycémique des patientes.

En cas d’inefficacité du régime, évaluée à chaque consultation ou lors de l’hôpital de jour; une insulinothérapie sera débutée : soit une injection sous cutané d’insuline NPH au coucher en cas d’hyperglycémie à jeun isolée ; soit d’une association d’analogue rapide et d’insuline semi-lente à chaque repas en cas de glycémie pré et post prandiale dépassant l’objectif.

*La stratégie de surveillance obstétricale est à adapter en fonction :*

- de **l’équilibre glycémique** obtenu ;
- de **la trophicité fœtale** : examen clinique, échographie pour estimation du poids fœtal (EPF) à 36 semaines d’aménorrhée à répéter à 38 semaines d’aménorrhée, si notion de macrosomie fœtale ;
- du **bien-être fœtal** : mouvements actifs et RCF.

*L’objectif de terme pour l’accouchement est :*

- **en cas de régime seul et d’objectifs glycémiques atteints** :
- fœtus eutrophe : 40 ou 41 semaines d’aménorrhée selon origine ethnique
- macrosomie : 38/39 SA
  - en cas de traitement par insuline:
- 38-39 SA

Dans tous les cas, si EPF > 4250 g et PA > 370 mm, l’accouchement sera réalisé par césarienne.

Dans les autres situations, la voie d’accouchement et le terme seront déterminés par l’obstétricien en charge de la patiente en fonction de la situation clinique. Le déroulement de l’accouchement et l’accueil des nouveau-nés sont réalisés selon les pratiques habituelles du service.

A l’arrivée en salle de naissance, un prélèvement sanguin sera réalisé chez la mère au moment de la pose de la perfusion. Il sera de 7 ml pour le dosage de l’hémoglobine glyquée (HbA1c) et de la fructosamine et de 7 ml pour le dosage de la leptine et de l’adiponectine.

Immédiatement après l’accouchement et la section du cordon ombilical, des prélèvements sanguins seront réalisés au niveau d’une des 2 artères du cordon ombilical : un prélèvement sur tube sec (7ml) pour la réalisation des dosages de peptide C, d’IGF1, de leptine, d’adiponectine et de ses isoformes (annexe 1), un prélèvement pour le dosage du pH réalisé sur l’automate dédié de la salle de naissance selon la technique habituelle, et un prélèvement pour le dosage de la glycémie qui sera réalisé au laboratoire de biochimie de l’hôpital de naissance après acheminement immédiat.

Les mensurations habituelles (poids, taille et périmètre crânien) des nouveau-nés sont colligées à la naissance, ainsi que le score d’Apgar à 5 et 10 minutes de vie, et un examen clinique complet est réalisé. Trois mesures répétées des plis cutanés au niveau tricipital, bicipital, supra-iliaque et sous-scapulaire seront effectuées entre le 1^er^ et le 3^ème^ jour de vie, lors de l’examen clinique systématique, à l’aide d’un adipomètre. Deux mesures répétées du périmètre crânien et de la taille seront également effectuées.

Les éléments relevés en rapport avec la morbidité néonatale sont : la prématurité (âge gestationnel < 37 SA), les malformations, les traumatismes obstétricaux (fractures, plexus brachial…), l’asphyxie périnatale définie par un score d’Apgar inférieur à 7 à 5 minutes de vie et un pH au sang de cordon < 7,2 mmol/L, la détresse respiratoire, c'est-à-dire toute difficulté respiratoire nécessitant une assistance au delà de 30 minutes de vie.

Un dépistage systématique des hypoglycémies néonatales est pratiqué chez les nouveau-nés de mère obèse ou diabétique et chez les nouveau-nés dystrophiques (poids de naissance ≤ 10^ème^ percentile ou ≥ 90^ème^ percentile). La mesure de la glycémie capillaire est réalisée à 1 heure de vie puis toutes les 3 heures pendant 24h minimum ou jusqu’à l’obtention de 3 glycémies capillaires consécutives supérieures à 3,3 mmol/L. La survenue d’une hypoglycémie < 2,5 mmol/L justifie une prise en charge adaptée selon un protocole préétabli. La mesure de la glycémie capillaire s’effectue au lit du bébé, à l’aide d’un glucomètre après ponction d’une goutte de sang au niveau du talon. Chez les nouveau-nés eutrophes (poids de naissance compris entre le 10^ème^ et le 90^ème^ percentile) de mère ayant un IMC pré-gestationnel compris entre 18,5 et 24,9 kg/m^2^ et pour lesquelles le dépistage du diabète est négatif, les glycémies capillaires ne seront pas contrôlées de façon systématique, comme cela est préconisé dans la pratique habituelle. Les épisodes d’hypoglycémie à révélation clinique seront relevés.

Les nouveau-nés présentant une pathologie néonatale qui nécessite une hospitalisation sont pris en charge selon le degré de gravité, dans l’unité de néonatologie de la Pitié-Salpêtrière, ou dans le service de réanimation ou de néonatologie de l’hôpital Trousseau (Paris 12^ème^).

La courbe de poids des nouveau-nés ainsi qu’un suivi précis de leur alimentation à la maternité seront relevés.

**4.5 Durée prévue de participation des personnes et description de la chronologie de l’essai:**

**Durée de participation de la personne à la recherche** = 9 mois (durée du suivi de la grossesse et la période néonatale c'est-à-dire par définition jusqu’à 28 jours de vie si nécessaire)

**Durée totale prévisionnelle de la recherche** = 36 mois.

**Durée d’inclusion** : 27 mois

**Chronologie de l’essai :**

| Mère | | | | | |  | Nouveau-né | |
| --- | --- | --- | --- | --- | --- | --- | --- | --- |
|  | Grossesse  Poids, TA, protéinurie tous les mois | | | | | Accouchement | Suites de couche | |
|  |  | | | | |  | 3 à 7 jours | |
| Date | 1^ère^ visite | 2^ème^ visite | 24-28 SA | 32 SA | 37-38 SA |  | J0 | J2-3 |
| Actes | IMC  Prise de TA  information  orientation | Consentement Inclusion  Poids  Prise de TA | Poids  Prise de TA | Echo : EPF |  | Poids, taille, PC  Apgar  Sang de cordon* | Examen  clinique | Examen clinique  Plis cutanés*  Poids taille, PC  Alimentation |
| Prélèvements |  | Glycémie  à jeun (pour les femmes obèses) | HGPO 75 g. | HGPO 75g*. | Glycémie à jeun* | **Mère:**  Fructosamine*  HbA1c*  Leptine*, Adiponectine*  **Nouveau-né (cordon):**  pH, glycémie*  Peptide C* IGF-1*  Leptine*Adiponectine* | Glycémies capillaires | |

* : examens spécifiques de la recherche

Les tests de dépistage du diabète (HGPO 75g) et les glycémies à jeun seront réalisés en ambulatoire. Les prélèvements à l’accouchement seront réalisés sur le lieu de la maternité (voir annexe 2).

**4.6 Description des règles d’arrêt définitif ou temporaire :**

**- de la participation d’une personne à la recherche :**

Seul le retrait du consentement peut entraîner l’arrêt de la participation d’une personne à la recherche.

**- d’une partie ou de la totalité de la recherche :**

Il n’y a pas de justification à l’arrêt de la recherche.

**4.7 Données à recueillir directement dans les cahiers d’observation :**

- Données maternelles recueillies à l’inclusion par l’interrogatoire et l’examen clinique
- Données du suivie de la grossesse :
- Relevé clinique mensuel : poids, TA, protéinurie
- Résultats et dates du dépistage du diabète : glycémies à jeun, valeurs de la glycémie après HGPO 75 g au 2^ème^ et 3^ème^ trimestre.
- Prise en charge du diabète : régime ou insuline
- Résultats des échographies et des rythmes cardiaques fœtaux : croissance fœtale, malformations, vitalité.
- Données néonatales :
- Apgar, pH au cordon
- Poids, taille, périmètre crânien
- Evènements périnataux : mortalité, malformations, traumatisme obstétrical, asphyxie périnatale, détresse respiratoire, prématurité
- Valeurs des glycémies des 24 premières heures et au-delà
- Hypoglycémies, valeurs
- Alimentation de la période néonatale
- Données biologiques :
- De la mère : HbA1c, fructosamine, leptine, adiponectine
- Du nouveau-né : glycémie au cordon, peptide C, IGF-1, leptine, adiponectine

**5. Sélection et exclusion des personnes de la recherche** :

**5.1 Critères d’inclusion des personnes qui se prêtent à la recherche :**

IMC ≥30 Kg/m2 ou entre 18,5 et 24,9 Kg/m2.

Femme enceinte consultant à la maternité avant 18 SA.

Age ≥ 18 ans et ≤ 40 ans

Grossesse unique

Absence de maladie constitutionnelle ou évolutive  autre que l’obésité : connectivites, maladies inflammatoires chroniques, épilepsie, pathologie psychiatrique, atteinte tumorale…

Recueil d’un consentement éclairé et écrit.

**5.2 Critères de non-inclusion des personnes qui se prêtent à la recherche :**

IMC < 18,5 Kg/m2 ou compris entre 25 et 29,9 Kg/m2.

Femmes enceinte consultant après 18 SA

Age < 18 ans ou > 40 ans

Grossesse multiple

Présence d’une maladie constitutionnelle ou évolutive autre que l’obésité.

Patientes ayant subi une chirurgie de l’obésité du type «by pass » de l’estomac ou une gastroplastie (anneau).

Obésité par maladie génétique ou secondaire à des lésions intra-crâniennes ou à une radiothérapie hypophysaire.

Pathologie endocrinienne non stabilisée.

Diabète connu avant la grossesse

Non affiliation à un régime de sécurité sociale (bénéficiaire ou ayant droit).

**5.3 Procédure d'arrêt prématuré de la recherche ou d'exclusion pour une personne de la recherche et procédure de suivi de la personne :**

Il s’agit d’une étude qui ne nécessite pas de procédure particulière en cas d’arrêt ou d’exclusion de la recherche.

**6. Traitement administré aux personnes qui se prêtent à la recherche :**

Aucun traitement n’est nécessaire à la réalisation de la recherche.

Tous les médicaments sont autorisés dans le cadre de la recherche, en dehors des traitements institués avant la grossesse dans le cadre d’une pathologie constitutionnelle ou évolutive et des médicaments potentiellement tératogènes.

**7. Evaluation de l'efficacité :**

**Non applicable**

8. Evaluation de la sécurité :

Il n’y a pas d’effets indésirables attendus spécifiques à la recherche. Les prélèvements maternels spécifiques seront réalisés à l’occasion d’autres prélèvements ou de la pose d’une perfusion. Le test d’HGPO supplémentaire réalisé au 3^ème^ trimestre ne présente pas de risque particulier. Le seul prélèvement spécifique à la recherche concernant le nouveau-né sera réalisé au niveau du cordon ombilical, après l’accouchement et section de celui-ci.

**9. Effets indésirables :**

Il n’y a pas d’effet indésirable attendu.

**10. Statistiques et gestion des données :**

**10.1 Description des méthodes statistiques prévues, y compris du calendrier des analyses intermédiaires prévues.**

Le plan expérimental de l’étude prévoit l’inclusion de deux groupes de femmes, obèses (IMC ≥30 kg/m^2^) et non obèses (18,5 ≤IMC< 25 kg/m^2^).

- *Analyses principales*

L’analyse principale est l’estimation du rôle de l’obésité maternelle sur la composition corporelle du nouveau-né. On cherchera notamment à différencier la part liée à l’obésité de celle liée au diabète. Les paramètres d’évaluation seront la somme des épaisseurs des plis cutanés, l’index pondéral et le poids. On utilisera un modèle d’analyse de covariance. Outre l’obésité et le diabète (non diabétique, diabète traité par régime seul et diabète traité par insuline), les facteurs de confusion potentiels seront introduits dans le modèle : âge maternel, hypertension pendant la grossesse, tabagisme, parité, sexe du nouveau-né, origine géographique, IMC paternel et l’âge gestationnel à la naissance, taille du père et de la mère, la maternité où la femme a été suivie. Les morts fœtales seront exclues de l’analyse.

- *Analyses secondaires*

- Les rôles respectifs de l’obésité et du diabète sur la fonctionnalité du tissu adipeux du nouveau-né, évaluée par le dosage de la leptine et de l’adiponectine réalisé au cordon, seront estimés par une analyse de variance.

- Le rôle des facteurs de croissance tels que IGF-1 et insuline (dont la sécrétion sera estimée par le dosage du peptide C) sur la composition corporelle du nouveau-né sera estimé au moyen d’une régression linéaire.

- Les relations entre les paramètres métaboliques maternels (leptine, adiponectine, hémoglobine glyquée HbA1c et fructosamine) et la masse grasse du nouveau-né (quantité et de fonctionnalité) d’une part et les facteurs de croissance du nouveau-né (peptide C et IGF-1) d’autre part seront étudiées au moyen d’une régression linéaire.

- Le taux de survenue d’une hypoglycémie dans les 48 heures suivant la naissance chez les nouveau-nés de mère obèse sera estimé avec son intervalle de confiance à 95%.

- Les taux d’évènements périnataux dans les deux groupes seront comparés au moyen du test exact de Fisher.

- *Calendrier des analyses*

Aucune analyse intermédiaire n’est prévue. L’analyse aura lieu 3 ans après le début de l’étude.

**10.2 Nombre prévu de personnes à inclure dans la recherche, et nombre prévu de personnes dans chaque lieu de recherches avec sa justification statistique.**

Les critères principaux de l’étude sont l’index pondéral et la somme des épaisseurs des plis cutanés à la naissance. En ce qui concerne les plis cutanés, l’étude de Sewell, Am J Obstet gyneco comparait 76 femmes en surpoids (IMC ≥ 25 kg/m^2^) à 144 femmes avec un IMC < 25 kg/m^2^. Les mesures des épaisseurs des plis cutanés au niveau tricipital et sous-scapulaire montraient une différence de 0,4 mm (écart-type de 0,95 mm) au niveau tricipital et une différence de 0,5 mm (écart-type de 1,2) au niveau sous-scapulaire, soit une taille d’effet pour les deux mesures de 0,42. Aucune donnée spécifique chez la femme obèse n’était présentée. Concernant l’index pondéral, nous ne disposons pas d’étude nous permettant de fonder des hypothèses pour un calcul de nombre de sujets. Nous souhaiterions pouvoir mettre en évidence une taille d’effet au moins égale à 0,3 pour ce critère.

Pour assurer à l’étude une puissance de 90% pour la mise en évidence d’une taille d’effet de 0,3, avec un risque de première espèce de 5%, l’analyse devra porter sur un total de 470 femmes (235 dans chacun des deux groupes). Le taux de femmes perdues de vue avant l’accouchement est estimé à 10%, celui de mort fœtale à 2% (les morts fœtales seront exclues des analyses portant sur les données anthropométriques). Les deux groupes étant appariés, on estime le taux de femmes non évaluables pour l’analyse principale à 24%. Il est donc nécessaire d’inclure 309 femmes dans chacun des deux groupes, soit un total de 618 femmes.

**10.3 Risque de première espèce**

Tous les tests seront bilatéraux et conduits au seuil de 5%.

**10.4 Critères statistiques d'arrêt de la recherche**

Aucun critère statistique d’arrêt n’est défini.

**10.5 Méthode de prise en compte des données manquantes, inutilisées ou non valides**.

Les sujets pour lesquels un critère d’évaluation ne sera pas recueilli seront exclus des analyses de ce critère.

**10.6 Gestion des modifications apportées au plan d'analyse de la stratégie initiale.**

Toute modification conséquente du plan d’analyse initial sera justifiée dans le rapport final de l’étude.

**10.7 Choix des personnes à inclure dans les analyses**

Les morts fœtales seront exclues des analyses portant sur les données anthropométriques mais prises en compte pour les analyses portant sur les évènements périnataux.

**10.8 Gestion des données**

La gestion des données de l’étude sera sous la responsabilité de l’Unité de Recherche Clinique Pitié-Salpêtrière/Charles Foix. Les données seront recueillies dans les cahiers d’observation électroniques. Le fichier de données sera déclaré à la CNIL.

**10.9 Responsable de l’analyse des données et logiciels de travail**

La planification de l’étude a été réalisée avec le logiciel N Query. Les analyses seront réalisées à l’aide du logiciel SAS version 8.2 (SAS Institute, Cary, NC), sous la responsabilité de l’Unité de Recherche Clinique Pitié-Salpêtrière/Charles Foix.

**11. Justification du budget :**

Demande des postes suivants répartis sur les deux centres investigateurs :

- Un ETP sage-femme dont les rôles seront :

- Repérage des patients éligibles pour l’étude en consultation, particulièrement les sujets témoins ;
- Information sur le projet de recherche aux patients éligibles pour l’étude avant recueil du consentement par le médecin ;
- S’assurer que les bilans nécessaires pour l’étude sont prescrits (HGPO et glycémie à jeun) ;
- Repérage des dossiers des femmes incluses pour leur identification rapide à l’arrivée en salle d’accouchement afin que le bilan soit réalisé.

- Un technicien de recherche clinique (4/5 temps) dont les rôles seront :

- S’assurer de l’organisation logistique des prélèvements (transport, stockage…)
- Remplissage des CRF

Coût des dosages hormonaux :

| Dosage | Nombre de points/patients | | Nombre de patients | Total points | Nombre de points/trousse | Nombre de trousses | Prix trousse HT | Total HT euros |
| --- | --- | --- | --- | --- | --- | --- | --- | --- |
| Leptine | 1 | 1 | 618 | 1236 | 80 | 15,45 | 390 | 6025,5 |
| Adiponectine et HMW | 1 | 1 | 618 | 1236 | 21 | 58,86 | 455 | 26 780 |
| IGF-1 |  | 1 | 618 | 618 | 38 | 16,26 | 475 | 7725 |
| Peptide C |  | 1 | 618 | 618 | 180 | 3,43 | 495 | 1 699,5 |
| Coût réactifs |  |  |  |  |  |  |  | **42 230** |

12. Droit d'accès aux données et documents source

Les personnes ayant un accès direct conformément aux dispositions législatives et réglementaires en vigueur, notamment les articles L.1121-3 et R.5121-13 du code de la santé publique (par exemple, les investigateurs, les personnes chargées du contrôle de qualité, les moniteurs, les assistants de recherche clinique, les auditeurs et toutes personnes appelées à collaborer aux essais) prendront toutes les précautions nécessaires en vue d'assurer la confidentialité des informations relatives aux médicaments expérimentaux, aux essais, aux personnes qui s'y prêtent et notamment en ce qui concerne leur identité ainsi qu’aux résultats obtenus. Les données collectées par ces personnes au cours des contrôles de qualité ou des audits seront alors rendues anonymes.

**13. Contrôle et assurance de la qualité**

**La recherche sera encadrée selon les procédures opératoires standards du promoteur.**

Le déroulement de la recherche dans les centres investigateurs et la prise en charge des sujets seront faites conformément à la déclaration d’Helsinki et les Bonnes Pratiques en vigueur.

Le déroulement de la recherche sera encadré selon la procédure opératoire standard du gestionnaire AP-HP. Les investigateurs de chaque centre s’engagent à recevoir les représentants nommés par l’AP-HP pour le contrôle de la qualité et les visites de conformité, le cas échéant.

14. Considérations légales et éthiques

Le promoteur est défini par la loi 2004-806 du 9 août 2004. Dans cette recherche, l'AP-HP est le promoteur et le Département de la Recherche Clinique et du Développement (DRCD) en assure les missions réglementaires.

**14.1 Demande d’autorisation auprès de l’AFSSAPS.**

Pour pouvoir démarrer la recherche, l’AP-HP en tant que promoteur doit soumettre un dossier de demande d’autorisation auprès de l'autorité compétente de l’AFSSAPS.

**14.2 Demande d’avis au Comité de Protection des Personnes**

En accord avec l'article L.1123-6 du Code de Santé Publique, le protocole de recherche sera soumis par le promoteur à un Comité de Protection des Personnes L'avis de ce comité sera notifié à l’autorité compétente par le promoteur avant le démarrage de la recherche.

**14.3 Modifications**

Le DRCD sera informé de tout projet de modification du protocole par l’investigateur coordonnateur.

**14.4 Déclaration CNIL**

La déclaration à la CNIL sera effectuée.

**14.5 Note d’information et Consentement éclairé**

Un double du (des) document(s) de consentement (annexe) sera remis à chaque patient. L’investigateur s’assurera que le sujet a bien compris les implications de la participation à l’essai et recueillera son consentement écrit (loi N°88-138, Art L.209.9).

Les formulaires d’information des patients et de consentement éclairé qui seront remis aux patients sont fournis en annexes 2a et 2b.

15. Traitement des données et conservation des documents et des données relatives à la recherche

Les documents spécifiques de la recherche seront archivés par toutes les parties pendant une durée de 15 ans après la fin de la recherche*.*

cet archivage indexé comportera :

- Les copies de courrier d’autorisation de l’AFSSAPS et de l’avis obligatoire du CPP
- Les versions successives du protocole (identifiées par le n° de version et la date de version),
- Les courriers de correspondance avec le promoteur,
- Les consentements signés des sujets avec la liste ou registre d’inclusion
- Le cahier d’observation complété et validé de chaque sujet inclus,
- Toutes les annexes spécifiques à l’étude,
- Le rapport final de l’étude
- La base de données ayant donné lieu à l’analyse statistique.

**16. Financement et assurance.**

Un financement est demandé au Programme Hospitalier de Recherche Clinique régional 2009. L’évaluation des dépenses est indiquée en annexe.

**17. Règles relatives à la publication**

Le rapport final de la recherche sera écrit en collaboration par le coordonnateur et le biostatisticien. seront premiers signataires des publications, les personnes ayant réellement participé à l’élaboration du protocole et son déroulement ainsi qu’à la rédaction des résultats. Ce rapport sera soumis à chacun des centres participants pour avis.

L’AP-HP sera propriétaire des données et aucune utilisation ou transmission à un tiers ne peut être effectuée sans son accord préalable.

L’Assistance Publique- Hôpitaux de Paris sera mentionnée comme étant le promoteur de la recherche biomédicale et comme soutien financier le cas échéant. les termes « Assistance Publique- Hôpitaux de Paris » apparaîtront dans l’adresse des auteurs.

**18. Liste des annexes**

**Annexe 1 :** Examens biologiques : modalités des prélèvements, de transport et de conservation. Techniques de dosage

**Annexes 2a et 2b :** Formulaires d’information des patients et de consentement éclairé.

**Annexe 1**

**Modalités de prélèvement, de transport et de conservation des prélèvements sanguins**

**Dosages de peptide C, d’hémoglobine A1c, de fructosamine, d’IGF-1, de leptine et d’adiponectine totale et formes de haut poids moléculaire (HMW)**

***Prélèvements***

**Le sang veineux des mères** sera prélevé sur 1 tube EDTA de 5 ml (hémoglobine A1c et fructosamine) et 1 tube sec de 7 ml (leptine et adiponectine totale et HMW).

**Le sang artériel du cordon** sera prélevé sur tube sec (7 ml) pour le dosage de peptide C, d’IGF1, de leptine et d’adiponectine totale et HMW.

***Transport des prélèvements depuis la consultation ou la salle de naissance***

Quelque soit le jour de la semaine, les prélèvements qui auront été réalisés entre 8h et 16h30, seront adressés par le pneumatique à la **réception commune des services de biochimie**, bâtiment de la Pharmacie, Groupe Hospitalier Pitié Salpêtrière ou au laboratoire de biochimie par coursier à l’hôpital Trousseau. Pour les prélèvements au-delà de 16h30, ils seront acheminés au laboratoire de biochimie de garde.

***Réception-aliquotage***

Au niveau de la réception commune des services de biochimie, les prélèvements seront enregistrés. Le prélèvement sur tube sec sera centrifugé et le sérum aliquoté en tubes Eppendorff, identifiés et datés. Le prélèvement sur tube à EDTA sera centrifugé, le plasma décanté et aliquoté en tube Eppendorff, identifié et daté (dosage de la fructosamine), et le culot cellulaire sera conservé dans le tube d’origine, identifié et daté (dosage de l’HbA1c). Tous les échantillons seront stockés à -30°C jusqu’à la réalisation des dosages.

***Transport des échantillons entre les hôpitaux***

Les dosages seront réalisés à la Pitié Salpêtrière. **L’hémoglobine A1c et la fructosamine seront pris en charge par le service de Biochimie Métabolique** (Pr B. Hainque), les **dosages de peptide C, d’IGF1, de leptine et d’adiponectine seront réalisés par le service de Biochimie Endocrinienne et Oncologique** (Pr J. Chambaz).

Les échantillons prélevés à Trousseau seront conservés au laboratoire de biochimie de Trousseau et transportés dans la carboglace tous les mois par coursiers vers les laboratoires concernés à la Pitié Salpêtrière.

***Les techniques de dosage sont les suivantes :***

*- Leptine* : RIA Linco Research, LABODIA, HL-81HK

*- Adiponectine* : technique ELISA, référence EK.MDP, Bülmann (France)

*- IGF 1* : RIA ALPCO, 22-IGF-R21

*- Peptide C* : IMMULITE, Siemens

- *Hémoglobine A1c*: échange d’ions CLHP Tosoh Biosciencs

- *Fructosamine* : réactif Roche

**Annexe 2a**

**Formulaire d’information des patients**

**Note d’information pour une personne participant à la recherche biomédicale intitulée**

**« Impact de l’obésité maternelle sur le développement fœtal et les évènements périnataux.**

**Analyse des déterminants de la croissance fœtale.»**

Promoteur : Assistance publique - hôpitaux de Paris

Madame,

Ce document est destiné à vous fournir des informations sur l’étude à laquelle nous vous proposons de participer. Avant de donner votre accord, il est important que vous le lisiez attentivement. L’investigateur ou le médecin qui vous recevra est à votre disposition pour répondre à toutes vos questions

Nous vous précisons que vous êtes en droit d’accepter ou non de participer à cette recherche.

Vous pouvez exercer à tout moment votre droit de retrait concernant votre participation.

Lorsque vous aurez lu cette note d’information et obtenu les réponses aux questions que vous vous posez en interrogeant le médecin, il vous sera proposé, si vous en êtes d’accord, de donner votre consentement écrit en signant le formulaire préparé à cet effet.

- **But de l’étude**

Nous vous proposons de participer à une recherche dont le but est de préciser les modifications de la composition corporelle du nouveau-né en fonction de la corpulence maternelle ; et d’analyser les conséquences observées chez le nouveau-né.

La croissance fœtale pendant la grossesse est dépendante de la sécrétion de certaines hormones, comme par exemple l’insuline, qui varient en fonction du poids de la mère et des troubles métaboliques survenant au cours de la grossesse.

Après la naissance, certaines variations hormonales peuvent entraîner des hypoglycémies qui nécessitent une surveillance et éventuellement une prise en charge rapide et adaptée.

- **Déroulement de l’étude**

Cette étude est réalisée de façon prospective, et étudie la composition corporelle et les taux hormonaux des nouveau-nés en fonction de la corpulence de leur mère.

Elle vise à comparer deux groupes de nouveau-nés : un groupe dont les mères présentent une obésité définie par un Indice de Masse Corporelle (Poids/Taille^2^) supérieur à 30 Kg/m^2^, à un groupe témoin dont les mères ont un Indice de Masse Corporelle compris entre 18 et 25 Kg/m^2^.

Vous êtes informées du déroulement de cette étude au début de votre grossesse, à la première visite à la maternité. Votre consentement est recueilli par l’obstétricien qui suivra votre grossesse.

Cette étude se déroule sur le lieu de la maternité. Il n’y a pas de risque particulier, ni de bénéfice direct attendus pour vous et votre enfant. Les seules différences par rapport à la prise en charge habituelle comportent : - le dépistage supplémentaire du diabète gestationnel vers 7 mois de grossesse ; - le dosage de la glycémie à jeun en fin de grossesse - les prélèvements sanguins à l’arrivée en salle de naissance - le prélèvement de sang au niveau du cordon ombilical, après sa section ; - la mesure des plis cutanés de votre bébé.

Cette recherche nécessite de prendre en compte certains évènements qui peuvent survenir pendant la grossesse, en particulier un diabète. Le dépistage du diabète est réalisé par le dosage de la glycémie après ingestion de 75g de sucre. Pour cette étude, il sera réalisé comme habituellement au cours du 2^ème^ trimestre de la grossesse (entre 5 et 6 mois), et une fois supplémentaire au 3^ème^ trimestre (vers 7 mois). La survenue d’un diabète justifie une prise en charge spécifique par l’équipe de diabétologie de l’hôpital. Afin de vérifier qu’il n’existe pas de modification de la glycémie après le dépistage du 7^ème^ mois, une glycémie à jeun sera réalisée au moment du prélèvement du bilan sanguin nécessaire à l’anesthésie péridurale de l’accouchement, ainsi qu’un prélèvement sanguin de 5 ml pour le dosage de l’insuline.

A l’arrivée en salle de naissance, au moment de la pose de la perfusion nécessaire pour l’accouchement, un prélèvement sanguin de 14 ml sera réalisé pour le dosage d’hormones et de composants reflétant le métabolisme en fin de grossesse. Ce dosage sera réalisé dans les semaines suivant le prélèvement qui ne sera pas conservé par la suite.

Après la section du cordon ombilical, la personne qui réalise l’accouchement recueillera 14 ml de sang au niveau de la partie du cordon rattachée au placenta. Ce geste n’entraîne aucune conséquence pour vous et pour votre enfant. Les dosages hormonaux nécessaires à la réalisation de cette étude seront effectués dans les semaines suivant le prélèvement qui ne sera pas conservé par la suite.

Un échantillon du placenta pourra être recueilli et conservé pendant 15 ans au laboratoire de Biochimie Endocrinienne et Oncologique (responsable Pr J Chambaz), situé dans le Bâtiment dit de la Pharmacie, GH Pitié-Salpêtrière. Il sera conservé pour des études ultérieures analysant les conséquences épigénétiques sur le placenta de la corpulence maternelle.

Pour dépister la survenue d’hypoglycémies néonatales, une mesure de la glycémie capillaire est réalisée durant les premières heures de vie chez les nouveau-nés dont les mères présentent une surcharge pondérale ou un diabète, et chez ceux qui ont un petit poids de naissance ou un gros poids de naissance, selon les recommandations habituelles. Celle-ci est effectuée toutes les 3 heures pendant les premières 24 heures ou jusqu’à l’obtention de trois glycémies consécutives normales. Elle est mesurée sur une goutte de sang prélevée après ponction au niveau du talon du nouveau-né. Les hypoglycémies sont traitées immédiatement par des apports adaptés de glucides.

Pour évaluer la composition corporelle de votre nouveau-né, une mesure des plis cutanés tricipital, bicipital, sous-scapulaire et supra-iliaque est réalisée lors de l’examen pédiatrique entre le 1^er^ et le 3^ème^ jour de vie. Celle-ci constitue un complément à l’examen clinique habituel et n’entraîne aucune contrainte particulière pour votre enfant.

**Durée de l’étude**

Cette recherche est organisée sur une durée de 3 ans.

La durée de votre participation à cette recherche correspond à la durée du suivi de votre grossesse et à la durée des soins pour votre bébé immédiatement après la naissance, c'est-à-dire habituellement la durée du séjour en maternité.

- **Promoteur**

L'Assistance publique - hôpitaux de Paris, qui organise cette recherche biomédicale en qualité de promoteur, a contracté une assurance conformément aux dispositions législatives, garantissant sa responsabilité civile et celle de tout intervenant auprès de la compagnie Gerling  France, dont l’adresse est 111 rue de Longchamp, 75116 Paris, par l’intermédiaire de BIOMEDIC INSURE (tel : 02 97 69 19 19) courtier en assurances*.*

- **Comité de Protection des Personne et AFFSAPS**

Cette recherche a reçu l’avis favorable du Comité de Protection des Personnes Ile-de-France ainsi que l’autorisation de mise en œuvre de l’autorité compétente de santé. Il est possible que cette recherche soit interrompue, si les circonstances le nécessitent, par le promoteur ou à la demande de l’autorité de santé.

- - **Confidentialité**

Dans le cadre de la recherche biomédicale à laquelle l’Assistance publique - Hôpitaux de Paris vous propose de participer, un traitement de vos données personnelles et celles de votre enfant va être mis en œuvre pour permettre d’analyser les résultats de la recherche au regard de l’objectif de cette dernière qui vous a été présenté.

A cette fin, les données médicales vous concernant, et les données relatives à vos origines géographiques seront transmises au promoteur de la recherche ou aux personnes ou sociétés agissant pour son compte, en France. Ces données seront identifiées par un numéro de code et vos initiales. Ces données pourront également, dans des conditions assurant leur confidentialité, être transmises aux autorités de santé françaises, à d’autres entités de l’Assistance publique – hôpitaux de Paris. Conformément aux dispositions de la CNIL (loi relative à l’informatique, aux fichiers et aux libertés), Vous disposez d’un droit d’accès et de rectification. Vous disposez également d’un droit d’opposition à la transmission des données couvertes par le secret professionnel susceptibles d’être utilisées dans le cadre de cette recherche et d’être traitées.

Vous pouvez également accéder directement ou par l’intermédiaire d’un médecin de votre choix à l’ensemble des données médicales vous concernant en application des dispositions de l’article L. 1111-7 du code de la santé publique. Ces droits s’exercent auprès du médecin qui le suit dans le cadre de la recherche et qui connaît son identité.

- - **Conditions de Participation**

Votre participation et celle de votre enfant à cette recherche biomédicale n'engendrera pour vous aucun frais supplémentaire.

Toutefois, pour pouvoir participer à cette recherche vous devez être bénéficiaire d’un régime de sécurité sociale.

Si vous n’acceptez pas le recueil des données concernant votre enfant, ou la réalisation du prélèvement de sang de cordon, vous de pouvez pas participer à cette recherche.

Cette étude n’entraîne pas de période d’exclusion si vous souhaitez participer à une autre recherche biomédicale à la fin de votre participation à cette recherche.

**Formulaire de consentement éclairé**

**Formulaire de Consentement de participation à une recherche biomédicale**

**pour les titulaires de l’autorité parentale sur un enfant**

Je, soussigné

M^e^, M^lle^, (*nom, prénom*) ……………………………………………

**Accepte librement et volontairement de participer à la recherche** **biomédicale intitulée** « **Impact de l’obésité maternelle sur le développement fœtal et les évènements périnataux. Analyse des déterminants de la croissance fœtale.**».

dont l’Assistance publique - hôpitaux de Paris est le promoteur et qui m’a été proposée par le

Docteur (*nom, prénom, téléphone*)……………………………………………………………………………..…. …………………………………………………….…, médecin dans cette recherche.

Etant entendu que :

- Le médecin qui m’a informé et a répondu à mes questions, m’a précisé que j’étais libre d'accepter ou de refuser de participer à cette recherche.

- Je pourrais avoir communication par le médecin au cours ou à l’issue de la recherche des informations qu’il détient concernant ma santé,

- J’ai bien compris dans la note d’information qui m’a été remise que pour pouvoir participer à cette recherche, je dois bénéficier d’un régime de sécurité sociale. Je confirme que c’est bien le cas,

- Je peux retirer à tout moment mon consentement à ma participation à cette recherche, et cela quelles que soient mes raisons et sans supporter aucune responsabilité. Je m’engage dans ce cas à en informer le médecin. Le fait de ne plus participer à cette recherche ne portera pas atteinte à mes relations avec ce médecin,

- Si je le souhaite, à son terme, je serai informée par le médecin des résultats globaux de cette recherche,

- Mon consentement ne décharge en rien le médecin et le promoteur de l’ensemble de leurs responsabilités et je conserve tous nos droits garantis par la loi.

*- J’ai bien noté que le droit d’accès prévu par la CNIL (loi du 6 janvier 1978 relative à l’informatique, aux fichiers et aux libertés (art. 39)) s’exerce à tout moment auprès du médecin qui me suit dans le cadre de la recherche et qui connaît mon identité. Je peux exercer mon droit de rectification et d’opposition auprès de ce même médecin, qui contactera le promoteur de la recherche.*

- *Je accepte/refuse* le recueil des données concernant mon enfant*
- *Je accepte/refuse* la réalisation d’un prélèvement de sang de cordon après la naissance de mon enfant.*
- *Je accepte/refuse* la conservation d’échantillons de placenta après la naissance de mon enfant.*
- *(*) rayer les mentions inutiles*

***la mère le père***

Fait à , le : Fait à , le :

Nom, prénom Nom, prénom

Signature Signature

**Rayer la mention inutile*

Signature du médecin qui atteste avoir pleinement expliqué à la personne signataire le but, les modalités ainsi que les risques potentiels de la recherche.

Date : Signature
